# Investigating hybrid nanoparticles for drug delivery in multi-stenosed catheterized arteries under magnetic field effects

**DOI:** 10.1038/s41598-024-51607-5

**Published:** 2024-01-12

**Authors:** Azad Hussain, Muhammad Naveel Riaz Dar, Warda Khalid Cheema, Rimsha Kanwal, Yanshuo Han

**Affiliations:** 1 Department of Mathematics, University of Gujrat, Gujrat, 50700 Pakistan; 2 School of Life and Pharmaceutical Sciences, Dalian University of Technology, Dalian, China

**Keywords:** Computational biology and bioinformatics, Mathematics and computing, Nanoscience and technology, Physics

## Abstract

This groundbreaking study pioneers the exploration of the therapeutic implications of a constant magnetic field simultaneously with hybrid nanoparticles on blood flow within a tapered artery, characterized by multiple stenosis along its exterior walls and a central thrombus, employing three-dimensional bio-fluid simulations. In addition, a magnetized catheter is inserted into the thrombus to increase the therapeutic potential of this novel method. The flow condition under consideration has applications in targeted medication distribution, improved medical device design, and improved diagnostics, as well as in advancing healthcare and biomedical engineering. Our investigation primarily aims to optimize blood flow efficiency, encompassing key parameters like pressure, velocity, and heat fluctuations influenced by diverse geometric constraints within the stenotic artery. Precise solutions are obtained through the finite element method (FEM) coupled with advanced bio-fluid dynamics (BFD) software. Hybrid nanoparticles and magnetic fields impacted pressure and velocity, notably reducing pressure within the stenosis. Convective heat flux remained uniform, while temperature profiles showed consistent inlet rise and gradual decline with transient variations. This approach promotes fluid flow, and convection within stenosed arteries, enhances heat transport, evacuates heat from stenotic regions, and improves heat dispersion to surrounding tissues. These findings hold promise for targeted therapies, benefiting patients with vascular disorders, and advancing our understanding of complex bio-fluid dynamics.

## Introduction

The flow of blood is constrained across damaged arteries with stenosis. This stenosis is caused by plaque accumulation on the arterial walls triggered by oil and fat deposits. This plaque accumulation can potentially cause multiple stenoses in severe cases. Because of multiple stenoses, the flow through these arteries is limited in several regions under certain conditions. Ponalagusamy^1^ investigated the flow through such narrowed tubes for the very first time in his Doctoral thesis. Mandal^2^ used the power law stream model to describe the unsteady blood flow via such tapering tubes. A numerical inquiry was conducted by Waqas et al.^3^ to examine the flow of nanofluid containing silver and gold nanoparticles introduced into a stenotic artery. Ponalagusamy’s^4^ analysis incorporates the different geometries of stenosis. Vonruden et al.^5^ reported a tentative examination of the flow of blood across a damaged artery with various stenosis. Javed et al.^6^ performed a comprehensive meta-analysis to investigate the impact of heterogeneous–homogeneous reactions within a sinusoidal wavy curved channel. Nadeem et al.^7^ used varying viscosity to theoretically describe the movement of nanotubes over an artery containing multiple stenoses. Misra et al.^8^ hypothesized blood flow as Casson fluid inside of an artery with multiple stenosis. Tang et al.^9^ investigated the flow characteristics of Au-nanofluid within a stenotic artery with porous walls, incorporating the Sisko model and accounting for the influence of viscous dissipation. Sreenadh et al.^10^ explored the blood flow through the artery with multiple stenoses, taking into account a mild example of multiple stenoses all along the artery’s length. There are three phases of stenosis development. It is modest at first (clogs approximately 5–15% of the artery area), then the obstruction rises to around 20 to 35% in the following stage and the stream retains laminar, although dissociation of flow and reverse stream occurs near the stenosis. Turbulence characterizes the last stage, with congestion exceeding 40% of the artery’s area^11^.

Another common kind of cardiovascular illness that can emerge as a consequence of stenosis worsening is thrombosis, which results from the establishment of a clot, which also barricades blood flow in the veins. Its progression can result in a variety of illnesses and ailments, including infarction, strokes, malignancy, and infections^12^. An arterial thrombus is a blood clot in the artery that can be deadly because it prevents blood from reaching vital organs. Several academics have recently focused on studying of bloodstream through arteries with thrombus in the center. Such blood clots (Thrombus) grow more commonly in sick arteries with tapering walls. As a result, the artery with severe stenosis seems to be more likely to develop a thrombus. This is a life-threatening case that almost limits the flow of blood and it has catastrophic implications. In this case, catheter insertion improves flow once again. This thin, empty pipe can be inserted into such problematic arteries to improve the flow. Doffin et al.^13^ inspected the flow issue with both scenarios (stenosis and thrombus) both analytically and experimentally. In this context, research into hybrid nanoparticles may lead to novel ways of avoiding thrombus development within stenosed arteries, thereby lowering the risk of life-threatening consequences such as embolism or stroke.

Catheters are frequent therapeutic and diagnostic instruments in modern healthcare. It is thin pipe put into the blood vessel to dispense medicine or to eliminate blood vessel blockages. The insertion of catheters helps to enhance blood flow to vital organs, and gasses (${\text{O}}_{2}\text{ and C}{\text{O}}_{2}$) in the transmission are frequently examined. A thin needle is located in the major artery at the arm, neck, or leg for clot removal, and a pliable wire is prolonged out in the artery through the lump. The catheter is directed along this line to the area of the thrombus to dissolve, split apart, and discharge it^14^. In the event of a congested or restricted artery, an inflatable balloon can be added from the catheter to unblock the conduit and improve blood flow. Catheters are also employed in disease diagnosis (e.g. X-ray angiography, intravascular ultrasound). Furthermore, in individuals who have a breathing problem or drastic acid/base disruption, a catheter is suitable for regular arterial blood gas analysis. When patients have a serious lung disease, the levels of carbon dioxide or oxygen in the blood must be checked more than three to four times each day. In this scenario, an artery catheter is utilized to take blood rather than repeatedly inserting a needle into the patient’s body^15^. Srivastava et al.^16^ numerically analyzed the macroscopic dual-phase hydrological model for blood circulation over a thin tube using a catheter. Nadeem et al.^17^ inspected blood flow through the catheterized artery with moderate stenosis numerically.

Choi^18^ was the first who invented nano-fluids. These are manipulated colloids constituted of nanoparticles and the base fluid. The nanoparticles are composed of metals, oxides and carbon nanotubes. These nanoparticles contain thermal conductivity and have a magnitude greater than the base fluid. Also, the sizes of nanoparticles are less than 100 nm. The preface of nanoparticles meliorates the performance of base fluids in heat transfer. These base fluids can be organic liquids, water, lubricants and oils, polymeric solutions, bio-fluids and a lot of other liquids. The nanoparticles have broad applications in medical like as bio-medicines, because of how nanoparticles collaborate with matter^19^. The dimensions and tailored shapes of hybrid nano-objects have diverted large-scale research due to their enormous concern in various exercises, including their optical characteristics and the capability for imaging. Nanoparticles may be utilized in the investigation, like mediators in optical, photoacoustic, in the delivery of drugs, as shippers capable of enhancing cancer disclosure to a therapeutic assistant, developing treatment fallout by continuance circulation times, preserving transported drugs from deterioration and increasing tumor assimilation. The blood-mediated nanoparticles consignment is the expanded and latest area in the progression of therapeutics and diagnostics. The characteristics of nanoparticles like surface chemistry, shape, and size can be handled to increase their objectives in human circulatory systems. This article offers a moving through a perfused area to determine how hybridized could assist in improving blood flow.

Mekheimer et al.^20^ used the combined action of a magnetic field and metallic nanoparticles on the micro-polar fluid flowing over an overlapped thrombosed artery like a blood circulation model. Atashafrooz et al.^21^ studied the simulation to analyze the coupled convective-radiative heat transfer phenomena in the flow of a hybrid nanofluid within an open trapezoidal enclosure, with a particular focus on assessing the influence of magnetic forces. Lubna et al.^22^ investigated the influence of nanofluids on an artery afflicted by stenosis and structural damage. A. Hussain et al.^23^ conducted a study to analyze the influence of heat transfer on time-dependent laminar fluid flow through a series of elliptic tubes. This investigation aimed to ascertain the extremes in velocity, pressure, and temperature results within the system. Waqas et al.^24^ explored heat transport characteristics in the context of nanofluid flow through a porous channel, while considering the impact of thermal radiation effects. Hybrid nano-fluids are relatively novel forms of nanofluid that may be created by suspending distinct types (two or more) of nanoparticles in the base fluid, and hybrid (compound) nanoparticles in the base fluid. A hybrid constituent is a substance that blends the physical and biochemical properties of many substances at the same time and delivers the properties in a uniform phase. Waqas et al.^25^ explored heat transfer phenomena in the context of hybrid nanofluid flow, taking into account the influence of thermal radiation, within the framework of a stretching sheet. The utilization of hybrid nanoparticles enables the exact delivery of therapeutic medicines to particular areas inside the arteries, optimizing medication concentrations precisely where they are required. Waqas et al.^26^ discovered a Proportional study of hybrid nanofluids with the Cattaneo-Christov convective heat flux model. Hybrid nanoparticles can help with minimally invasive therapies, which reduces the need for invasive surgeries, increasing patient outcomes and shortening recovery periods. Increased accuracy in medication administration can result in lower drug doses while retaining therapeutic efficacy, potentially lowering the likelihood of side effects. Waqas et al.^27^ explored a numerical and computational simulation to investigate blood flow with heat transfer in a stenotic artery, incorporating the use of hybrid nanofluids. The physicochemical characteristics of synthesized hybrid nanostructures are exceptional since they are not present in the separate components. A substantial chunk of investigation has been conducted on the characteristics of these composite materials^28^, and hybrid substances carbon nanotubes (CNTs) are being utilized in chemical devices, spectroscopy, nanofibers, and other applications^29^, however, the application of these composite nanomaterials in nanofluids has not established. Research on hybrid nanofluids is still in its early stages, with more scientific and theoretical studies yet to be conducted. Nazir et al.^30^ examined finite element simulations to analyze the behavior of a hybrid nano-Carreau Yasuda fluid under the influence of Hall and ion slip forces over a rotating heated porous cone. Ahmed and Nadeem^31^ studied the presence of minor stenosis plaques in the presence of several types of nanoparticles composed with copper (Cu), aluminum (Al2O3), and titanium dioxide (TiO2).

Bio-magnetic hydrodynamics is a novel field of fluid mechanics that studies the fluid dynamics of bio-fluids in the existence of magnetic fields. Several scientists are eager to learn more about the impact of magnetic flux on the blood flow. Nevertheless, Haik et al.^32^ discovered bio-magnetic fluid dynamics. Akbar et al.^33^ discovered 3-D magnetohydrodynamic (MHD) viscous flow, considering the effects of thermal radiation and viscous dissipation. Biological fluids are Ferro-fluids, which are magnetic fluids that do not carry electricity. Ferro-fluids are used in a variety of applications, including pharmaceuticals, and anti-tumor drug haulers. Researchers are interested in investigating basic bio-magnetic hydrodynamics flow problems because of the innumerable major applications in biomedical engineering and biological sciences, like the advancement of magnetic materials for cell parting, targeted therapies transfer employing magnetic nanoparticles as medication delivery transporters, magnetic help combat and cancer therapy instigating magnetic hypoglycemia, minimizing blood loss during surgical treatment, and incitement of occlusive disease. Another notable implementation of these liquids is in chemotherapy. Tang et al.^34^ numerically studied the magnetized flow of Powell–Eyring hybrid nanomaterial, incorporating variable heat transfer phenomena in the presence of artificial bacteria and explored potential applications in tumor removal and the destruction of cancer cells. Magnetic field effects can improve the targeting of drug-loaded nanoparticles to stenosed areas, resulting in more efficient drug administration and fewer systemic adverse effects. Understanding how hybrid nanoparticles behave in multi-stenosed catheterized arteries in the presence of a magnetic field might help to create personalized medication delivery systems customized to particular patients’ vascular problems. The medicine is coated with magnetized nanoparticles and administered adjacent to the malignance in the treatment. The drug is immersed by the malignant tumor using an extravagant magnetic field focused around the tumor’s midpoint. This method diminishes the adverse effects of anticancer drugs. This approach has primarily been tested on tiny animals. Recently, several studies reported the use of such a technology for human therapy in cases when the tumor is close to the skin^35^.

The previously collected research work is extensively examined and it is found that the consequence of hybrid nanoparticles and uniform magnetic field on the bloodstream in defective arteries with several stenoses on the exterior walls and a thrombus (clot) in the middle has not yet been studied using a catheter through three-dimensional computational simulation. This type of investigation is carried out to cover this void in the literature by getting the prevailing nonlinear Navier Stokes equations. These findings will help future research in analyzing the use of magnetic resonance imaging (MRI) and catheterization for circulation, which are both important radiological tests for atherosclerosis^36^. This technique improves fluid dynamics and convection inside restricted arteries, optimizing heat transfer, allowing for effective heat removal from stenotic sites, and enhancing heat dispersion into neighboring tissues. The findings of this study might aid in the development of new medical devices that use magnetic fields to optimize medicine delivery within stenosed arteries. Finally, this discovery might pave the way for the creation of new treatment modalities that use the potential of hybrid nanoparticles and magnetic fields to address difficult vascular diseases.

## Materials and methods

**Figure 1:**
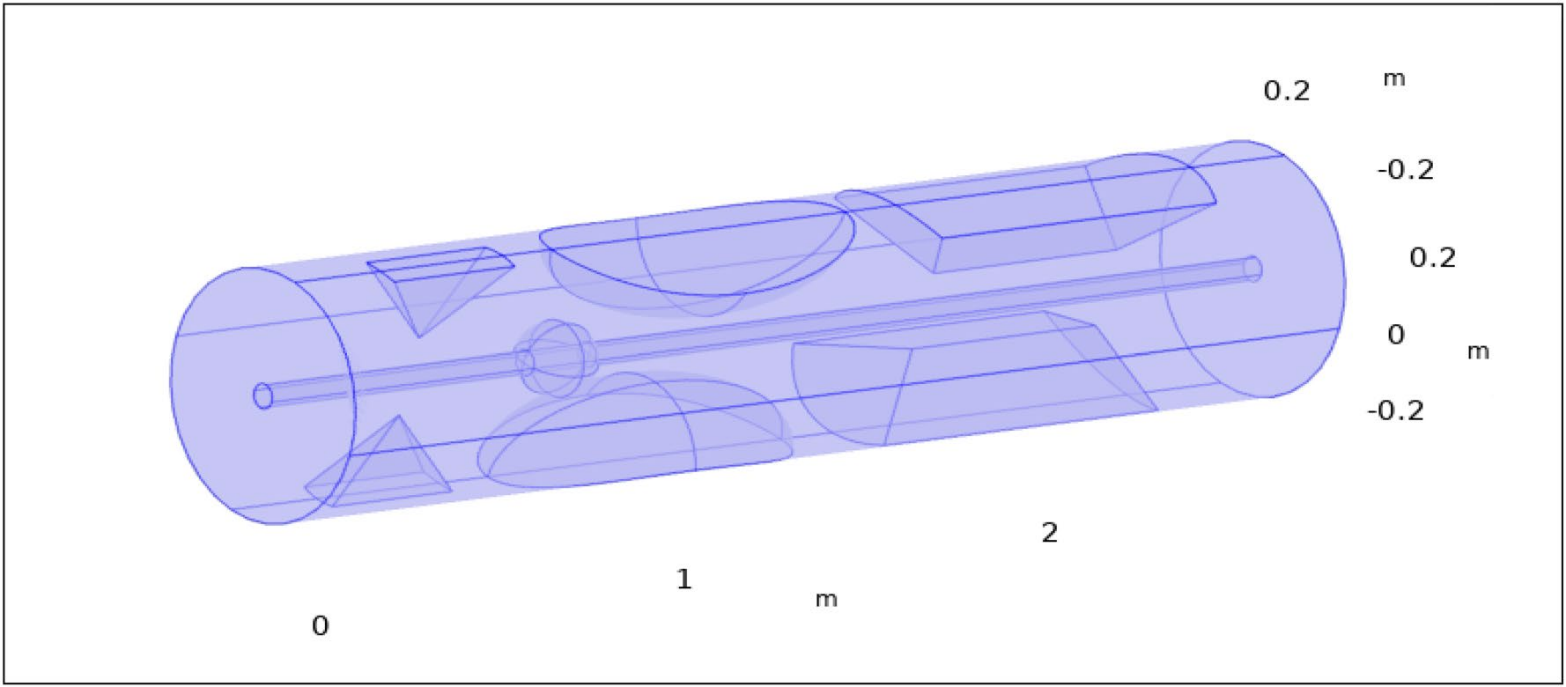
3-D visualization of catheterized arteries with intricate stenosis and thrombotic clots.

Let us look into the computational simulation for the unsteady, incompressible, two-dimensional, and laminar blood flow across a circular artery of finite Length L 2.7 m, that is made up of two co-axial cylinders: the outer cylinder with a radius of 0.3 m, which is an axisymmetric having multiple stenoses, and an inner pipe (catheter) with radius 0.03 m containing hybrid nanoparticles (silver and gold) which are injected in the artery as a drug, passing through a thrombus (blood clot) developed at the center of the stenosed artery. We seek to emulate blood artery flow and electrical characteristics. Thus a uniform magnetic field is given to the catheter to examine its effect on blood flow. Blood is diamagnetic because it mostly comprises water (plasma) and red blood cells. The relative permeability of diamagnetic materials is extremely near to, or slightly lower than, 1. In the majority of applications, the magnetic properties of blood are thought to be insignificant. So we inject hybrid nanoparticles to enhance the magnetic characteristics which can determine relative permeability. The electrical conductivity of the blood is taken as 0.70 ${\text{S}}/{\text{m}}$ and its relative permittivity is $5\times {10}^{3}$. The tube’s wall has a relative permittivity and conductivity of $1.63\times {10}^{3}$ and 0.31 ${\text{S}}/{\text{m}}$ respectively^37^. As illustrated in Fig. 1, it is preferable to work with the cylindrical coordinates (r, θ, z), where the z-axis is considered along the direction of the horizontal artery and θ and r the circumferential and radial directions, correspondingly. The flow is taken along the axial direction z and r is perpendicular to the flow.

Correspondingly, the equations of an unsteady, viscous, and incompressible, hybrid nano-fluid along a catheterized stenotic artery having thrombosis and applying a magnetic field to the catheter and thrombus are given as^38^:

1 $$\rho \frac{\partial \mathbf{u}}{\partial t}+\rho \left(\mathbf{u}\cdot \nabla \right)\mathbf{u}=\nabla \cdot \left[-p\mathbf{I}]+\nabla [{\varvec{K}}\right]+{\varvec{F}},$$

where $$\mathbf{K}={\upmu (\nabla \mathbf{u}+(\nabla (\mathbf{u}))}^{{\text{T}}})$$.

2 $$\rho \nabla \cdot \left(\mathbf{u}\right)=0, \left(\mathrm{Incompressible\, flow}\right),$$

3 $$\rho {{\text{C}}}_{{\text{p}}}\frac{\partial {\text{T}}}{\partial t}+\rho {C}_{p}\mathbf{u}\cdot \nabla {\text{T}}+\nabla \cdot {\varvec{q}}={Q}_{p}+{Q}_{vd}+Q.$$

The underlying relationships comprise the Eq. (6).

4 $${\varvec{q}}=-k\nabla T, Q=0, { Q}_{vd}=\tau .\nabla u, { Q}_{p}={\alpha }_{p}T\left(\frac{\partial p}{\partial t}+u\nabla p\right), {\alpha }_{p}=-\frac{1}{p}\frac{\partial p}{\partial t},$$

where $$\tau =-pI+\mu {A}_{1}$$ and $$trace\left(\tau \cdot \nabla {\text{u}}\right)=\tau .$$

The equations of the functional magnetic field are given as:

5 $$\nabla \times H=J, B=\nabla \times A, J=\sigma E+{J}_{e}, E=-\frac{\partial A}{\partial t}.$$

$\nabla \times H=J$ is Ampère’s law for magneto-statics. It states that the curl of the magnetic field intensity (H) is equal to the current density (J). $B=\nabla \times A$ relates the magnetic field (B) to the curl of the vector potential (A). $J=\sigma E+{J}_{e}$ describes the current density (J) in terms of two components: conduction current density $\sigma E$ and any additional current density ${J}_{e}$. The first term $\sigma E$ represents the conduction of electric current due to the electric field (E) and the electrical conductivity (σ) of the material. The second term ${J}_{e}$ accounts for any other sources of current density that might be present. $E=-\frac{\partial A}{\partial t}$ represents Faraday’s law of electromagnetic induction. It states that the electric field E is equal to the negative rate of change of the vector potential A concerning time t. This law indicates that a changing magnetic field induces an electric field, which is a foundational principle in electromagnetic theory.

## Boundary conditions

Boundary conditions at arteries wall can be prescribed as^27^.

### Inlet boundary condition

At the inlet of the artery, blood velocity is zero. The regulation of arterial pressure can be influenced by the velocity at the intake and the inflow rate.

The boundary condition equation at the inlet is expressed as follows:

6 $${u}_{r }\left({\text{r}},\mathrm{ z},\mathrm{ t}\right)= -{U}_{0}\mathbf{n}.$$

### Outlet boundary condition

To enhance the realism of the simulations, we included the blood supply model’s pressure at the outflow, which is located on the other side of the inlet border. The outlet equation is as follows:

7 $$\left[-{\text{P}}\mathbf{I}+\mathbf{K}\right]\mathsf{n} = -\widehat{{{\text{P}}}_{0}}\mathsf{n},$$

$$\widehat{{{\text{P}}}_{0}} \le {{\text{P}}}_{0}.$$

### Wall boundary condition

Due to the viscous characteristics of blood, it exhibits adherence to the walls without penetrating them, and a no-slip condition is assumed. Consequently, the equation at the wall is expressed as follows:

8 $${u}_{r }=0, {u}_{z}=0.$$

### Thermal insulation condition

All boundaries surrounding the geometry are thermally insulated, and the equation governing thermal insulation is presented as follows:

9 $$-\mathbf{n}.\mathbf{q}= 0.$$

### Heat flux condition

A general inward heat flux of $1200\text{ Wm}^{2}$ is supplied to the inlet of the cylinder. The main equation of heat flux is specified as:

10 $$-\mathbf{n} \cdot \mathbf{q}= {\mathbf{q}}_{0}.$$

Boundary conditions at the catheter wall are given as follows.

### Magnetic insulation condition

The equation of magnetic insulation is given by

11 $$\mathsf{n}\times \mathsf{A}=0.$$

## Mathematical modeling

The equations for momentum, mass, and energy for the specified velocity field $V=\left({v}_{r }\left(r, \theta , z, t\right),{v}_{\theta }\left(r, \theta , z, t\right),{v}_{z}\left({\text{r}},\uptheta ,\mathrm{ z},\mathrm{ t}\right)\right)$ is as follows^39^.

**Continuity equation**

12 $$\frac{\partial {v}_{r }}{\partial r}+\frac{1}{r}{v}_{r }+\frac{1}{r}\frac{\partial {v}_{\theta }}{\partial \theta }+\frac{\partial {v}_{z }}{\partial z}=0.$$

**Momentum equations**

13 $${\rho }_{hnf}\left( \frac{\partial {{\text{v}}}_{\mathrm{r }}}{\partial {\text{t}}}+ {{\text{v}}}_{\mathrm{r }} \frac{\partial {{\text{v}}}_{\mathrm{r }}}{\partial {\text{r}}}+\frac{{{\text{v}}}_{\uptheta }}{{\text{r}}} \frac{\partial {{\text{v}}}_{\mathrm{r }}}{\partial\uptheta }-\frac{{{{\text{v}}}_{\uptheta }}^{2}}{{\text{r}}}+ {{\text{v}}}_{\mathrm{z }} \frac{\partial {{\text{v}}}_{\mathrm{r }}}{\partial {\text{z}}}\right)=-\frac{\partial p}{\partial r}+\frac{1}{r}\frac{\partial \left(r{\mathcal{T}}_{rr}\right)}{\partial r}+\frac{1}{r}\frac{\partial \left({\mathcal{T}}_{r\theta }\right)}{\partial \theta }-\frac{{\mathcal{T}}_{\theta \theta }}{r}+\frac{\partial \left({\mathcal{T}}_{rz}\right)}{\partial z},$$

14 $${\rho }_{hnf}\left( \frac{\partial {{\text{v}}}_{\uptheta }}{\partial {\text{t}}}+ {{\text{v}}}_{\mathrm{r }} \frac{\partial {{\text{v}}}_{\uptheta }}{\partial {\text{r}}}+\frac{{{\text{v}}}_{\uptheta }}{{\text{r}}} \frac{\partial {{\text{v}}}_{\uptheta }}{\partial\uptheta }-\frac{{{\text{v}}}_{{\text{r}}}{{\text{v}}}_{\uptheta }}{{\text{r}}}+ {{\text{v}}}_{\mathrm{z }} \frac{\partial {{\text{v}}}_{\uptheta }}{\partial {\text{z}}}\right)=-\frac{1}{{\text{r}}}\frac{\partial {\text{p}}}{\partial\uptheta }+\frac{1}{{{\text{r}}}^{2}}\frac{\partial \left({{\text{r}}}^{2}{\mathcal{T}}_{\mathrm{\theta r}}\right)}{\partial {\text{r}}}+\frac{1}{{\text{r}}}\frac{\partial {\mathcal{T}}_{\mathrm{\theta \theta }}}{\partial\uptheta }+\frac{\partial {\mathcal{T}}_{\mathrm{\theta z}}}{\partial {\text{z}}},$$

15 $${\rho }_{hnf}\left( \frac{\partial {{\text{v}}}_{\mathrm{z }}}{\partial {\text{t}}}+ {{\text{v}}}_{\mathrm{r }} \frac{\partial {{\text{v}}}_{\mathrm{z }}}{\partial {\text{r}}}+\frac{{{\text{v}}}_{\uptheta }}{{\text{r}}} \frac{\partial {{\text{v}}}_{\mathrm{z }}}{\partial\uptheta }+ {{\text{v}}}_{\mathrm{z }} \frac{\partial {{\text{v}}}_{\uptheta }}{\partial {\text{z}}}\right)=-\frac{\partial {\text{p}}}{\partial {\text{z}}}+\frac{1}{{\text{r}}}\frac{\partial \left({\text{r}}{\mathcal{T}}_{{\text{rz}}}\right)}{\partial {\text{r}}}+\frac{1}{{\text{r}}}\frac{\partial \left({\mathcal{T}}_{\mathrm{\theta z}}\right)}{\partial\uptheta }+\frac{\partial \left({\mathcal{T}}_{{\text{zz}}}\right)}{\partial {\text{z}}},$$

where $$\mathcal{T}=\left({\left(\nabla \left(U\right)\right)}^{T}+\nabla \left(U\right)\right)$$.

**Energy equation**

16 $${(\rho {C}_{p})}_{hnf}\left(\frac{\partial T}{\partial t}+{v}_{r }\frac{\partial T}{\partial r}+\frac{{v}_{\theta }}{r}\frac{\partial T}{\partial \theta }+{v}_{z }\frac{\partial T}{\partial z}\right)={{\text{k}}}_{hnf}\left[\frac{1}{r}\frac{\partial }{\partial r}\left(r\frac{\partial T}{\partial r}\right)+\frac{1}{{r}^{2}}\frac{{\partial }^{2}T}{\partial {\theta }^{2}}+\frac{{\partial }^{2}T}{\partial {z}^{2}}\right]+{\mu }_{hnf}\varphi .$$

In the context of compressible flow, the dissipation function within cylindrical coordinates is represented as follows:

$$\varphi =2{\left( \frac{\partial {v}_{r }}{\partial r}\right)}^{2}+2{\left(\frac{1}{r}\frac{\partial {v}_{\theta }}{\partial \theta }+\frac{{v}_{r }}{r}\right)}^{2}+2{\left( \frac{\partial {v}_{z}}{\partial z}\right)}^{2}+{\left( \frac{\partial {v}_{\theta }}{\partial r}-\frac{{v}_{\theta }}{r}+\frac{1}{r} \frac{\partial {v}_{r }}{\partial \theta }\right)}^{2}+{\left(\frac{1}{r}\frac{\partial {v}_{z }}{\partial \theta }+\frac{\partial {v}_{\theta }}{\partial z}\right)}^{2}+{\left( \frac{\partial {v}_{r }}{\partial z}+\frac{\partial {v}_{z }}{\partial r}\right)}^{2}.$$

After relating the velocity vector U = [${v}_{r}(r, z, t),0, {v}_{z }(z, r, t)]$, and uniform magnetic field the above equation reduces to the following equations

17 $$\frac{\partial {v}_{r}}{\partial r}+\frac{{v}_{r}}{r}+\frac{\partial {v}_{z }}{\partial z}=0,$$

18 $$\left(\frac{\partial {v}_{r }}{\partial t}+ {v}_{r }\frac{\partial {v}_{r }}{\partial r}+ {v}_{z }\frac{\partial {v}_{r }}{\partial z}\right)=-\frac{1}{\rho }\frac{\partial p}{\partial r}+{\nu }_{hnf}\left(\frac{{\partial }^{2}{v}_{r}}{\partial {r}^{2}}+\frac{1}{r}\right. \frac{\partial {v}_{r }}{\partial r}+\frac{{\partial }^{2}{v}_{r}}{\partial {z}^{2}}\left.-\frac{{v}_{r}}{{r}^{2}}\right)+g({\rho \gamma )}_{hnf}\alpha \left(T-{T}_{0}\right)-\sigma {{B}_{0}}^{2}{v}_{r },$$

19 $$\frac{\partial p}{\partial \theta }=0,$$

20 $$\left(\frac{\partial {v}_{z }}{\partial t}+ {v}_{r }\frac{\partial {v}_{z }}{\partial r}+ {v}_{z }\frac{\partial {v}_{z}}{\partial z}\right)=-\frac{1}{\rho }\frac{\partial p}{\partial z}+{\nu }_{hnf}\left(\frac{{\partial }^{2}{v}_{z}}{\partial {r}^{2}}+\frac{{\partial }^{2}{v}_{z}}{\partial {z}^{2}}+\frac{1}{r}\frac{\partial {v}_{z}}{\partial r}\right)+g({\rho \gamma )}_{hnf}\alpha \left(T-{T}_{0}\right)-\sigma {{B}_{0}}^{2}{v}_{z },$$

21 $${(\rho {C}_{p})}_{hnf}\left(\frac{\partial T}{\partial t}+{v}_{r }\frac{\partial T}{\partial r}+{v}_{z}\frac{\partial T}{\partial z}\right)={K}_{hnf}\left(\frac{1}{r}\frac{\partial T}{\partial r}+\frac{{\partial }^{2}T}{\partial {r}^{2}}+\frac{{\partial }^{2}T}{\partial {z}^{2}}\right)+{Q}_{0}.$$

In this formulation, ${\rho }_{hnf}$ represents the density, ${\nu }_{hnf}$ denotes the kinematic viscosity of the hybrid nano-fluid, and T signifies the absolute temperature. Additionally, the specific heat capacity and thermal conductivity of nanoparticles are represented as ${\left(\rho {C}_{p}\right)}_{hnf}$ and ${K}_{hnf}$, respectively.

The following are the thermo-physical characteristics of hybrid nanoparticles^40^ (Table 1):

**Table 1:** Numerical data of blood, gold, and silver nanoparticles^41^.

Property	Heat capacity $\left({\text{J}}{\text{ K}}^{-1} {\text{kg}}^{-1}\right)$	Thermal conductivity $\left({\text{W m}}^{-1} {k}^{-1}\right)$	Dynamic viscosity $\left({\text{Nm}}^{-2}\text{ s}\right)$	Density $\left({\text{kg m}}^{-3}\right)$
Blood	3746	0.52	0.003	1063
Gold (Au)	129	310	0.00464	19,300
Silver (Ag)	235	429	0.005	10,500

22 $$\left.\begin{array}{c}{\rho }_{hnf}= \left(1-{\phi }_{2}\right)\left( \left(1-{\phi }_{1}\right)\right.{\rho }_{f}+{\phi }_{1} {\rho }_{{s}_{1}})+{\phi }_{2}{\rho }_{{s}_{2}}\\ {(\rho {C}_{p})}_{hnf}=\left(1-{\phi }_{2}\right){(\left(1-{\phi }_{1}\right)\rho {C}_{p})}_{f}+{\phi }_{1}{(\rho {C}_{p})}_{{s}_{1}})+{\phi }_{2}{(\rho {C}_{p})}_{{s}_{2}}\\ {\mu }_{hnf}= \frac{{\mu }_{f}}{{\left(1-{\phi }_{1}\right)}^{2.5}{\left(1-{\phi }_{2}\right)}^{2.5}}\\ \frac{{K}_{hnf}}{{K}_{f}}= \left\{\frac{{k}_{{s}_{1}+}2{k}_{f}-2{\phi }_{1}\left({k}_{f}-{k}_{{s}_{1}}\right)}{{k}_{{s}_{1}+}2{k}_{f}+{\phi }_{1}\left({k}_{f}-{k}_{{s}_{1}}\right)}\times \frac{{k}_{{s}_{2}+}2{k}_{nf}-2{\phi }_{2}\left({k}_{nf}-{k}_{{s}_{2}}\right)}{{k}_{{s}_{2}+}2{k}_{nf}+{\phi }_{2}\left({k}_{nf}-{k}_{{s}_{2}}\right)}\right\}\end{array}\right\}.$$

## Computational mesh and numerical methodology

**Figure 2:**
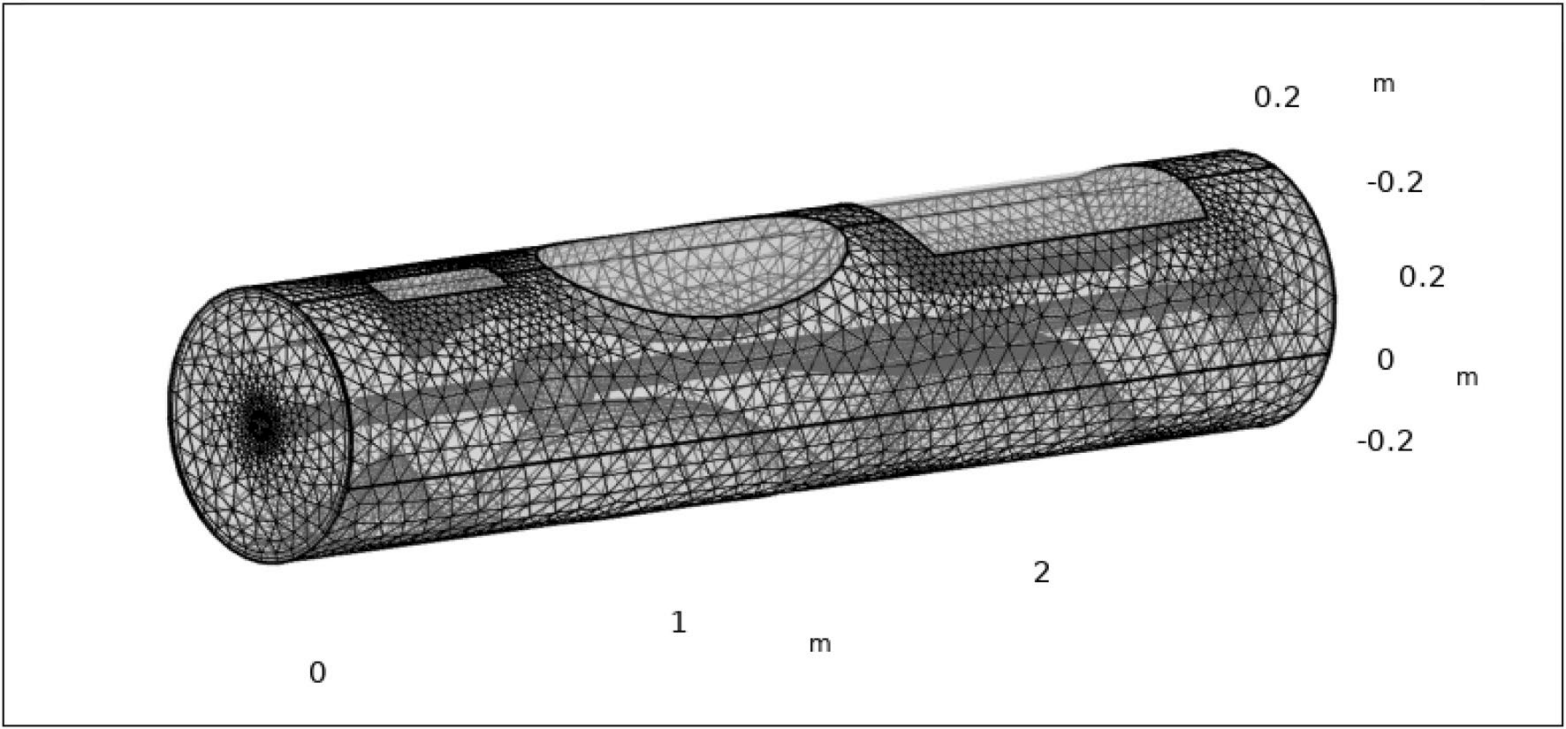
Exploring the intricacies of complex geometry through its elaborate finite element mesh.

We used a numerical technique based on finite element discretization because no other solutions were available. After linearization, we used an iterative direct-type solver in COMSOL Multi-physics software to solve the nonlinear algebraic problem. Grid refinement was accomplished by employing normal grid with comparable degrees of freedom, as seen in Fig. 2. To achieve a better degree of accuracy in our results, the simulation procedure, statistical data, and all graphs were only constructed using the normal element mesh. We chose this particular mesh arrangement to acquire the most dependable and exact results for our investigation. The finite element discretization of the geometry follows the same principles as the discretization of the system’s mathematical model, the result is approximated as continuous across each couple of points in the mesh. It is noted that the mesh is extra rectified in the stenotic section, as can be observed by the stenotic area, and less rectified while far distant from the stenosis, as seen in Fig. 2. The elucidation of the size of the mesh and other information about the mesh stats is given in Tables 2 and 3 respectively.

**Table 2:** Description of mesh dimensions.

Geometric constituents units	Bounds	Geometric constituents units	Bounds
Illustrate for	Fluid dynamics	Least element size	0.031
Perseverance of contracted section	0.7	Determination of confined zone	0.7
Extreme constituent growth rate	1.15	Curvature factor	0.6
Maximum component size	0.104	Predefined size	Normal

**Table 3:** Description of mesh dimensions.

Characteristics	Values
Number of elements	279,109
Tetrahedrons	250,001
Prisms	28,016
Triangles	16,424
Quads	172
Pyramids	1092
Vertex elements	70
Edge elements	1620
Normal element quality	0.6633
Least element quality	0.07794
Mesh vertices	58,620
Element volume ratio	6.445E−4
Mesh volume	${0.6429\text{ m}}^{3}$

The mesh enables the numerical solution of the governing equations by separating the computational domain into smaller elements or cells. The equations are solved on this discrete mesh to approximate the behavior of the physical system under study. The mesh plays a substantial role in achieving convergence of the numerical solution. The mesh is designed with a normal element size, and considerations are made to maintain mesh quality by evaluating the skewness measure. The partial mesh consists of mesh vertices, comprising various types of vertex elements, edge elements, quadrilateral elements, triangular elements, prism elements, tetrahedron elements and many other aspects. The description of all the mesh these mesh dimensions is specified in Table 3.

## Results and outcomes

The effect of uniform magnetic field and hybrid nanoparticles (silver and gold) on blood flow in defective arteries with several stenosis on the exterior walls and a thrombus (clot) in the middle of the artery using a catheter through three-dimensional computational simulation is deliberate in this research. The findings indicate that the shape of stenosis and thrombus in the middle of an artery is the key cause of elevated blood pressure at arterial walls. The outcomes proffered here validate the bloodstream features of the arteries with stenosis and thrombosis. The inclusion of hybridized nanoparticles altered the physical possessions of blood, like density, heat capacity, thermal conductivity, and dynamic viscosity, which influenced the simulation results.

### Surface and contour velocity preeminence

**Figure 3:**
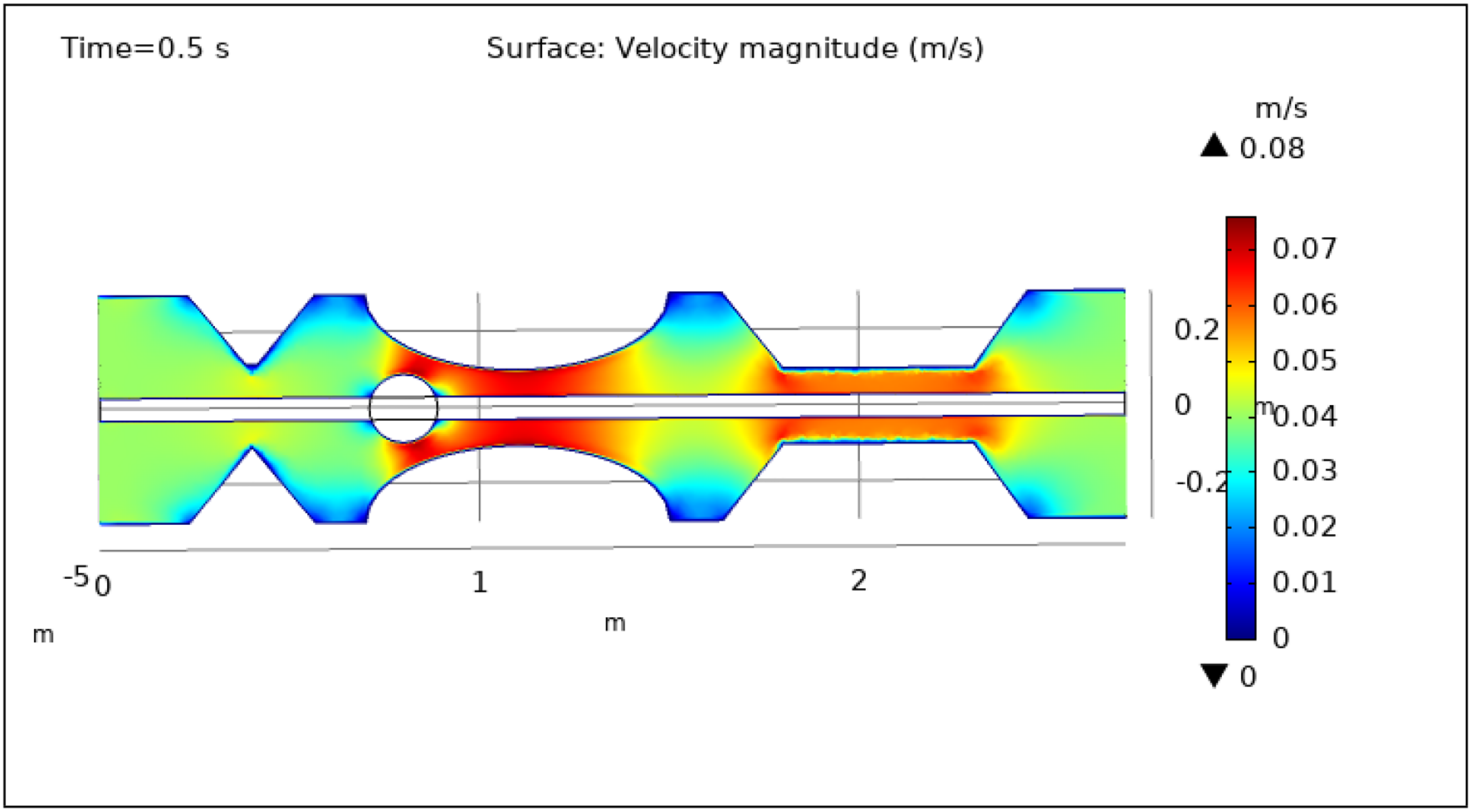
Velocity profile for $t=0.5\text{ s}$.

**Figure 4:**
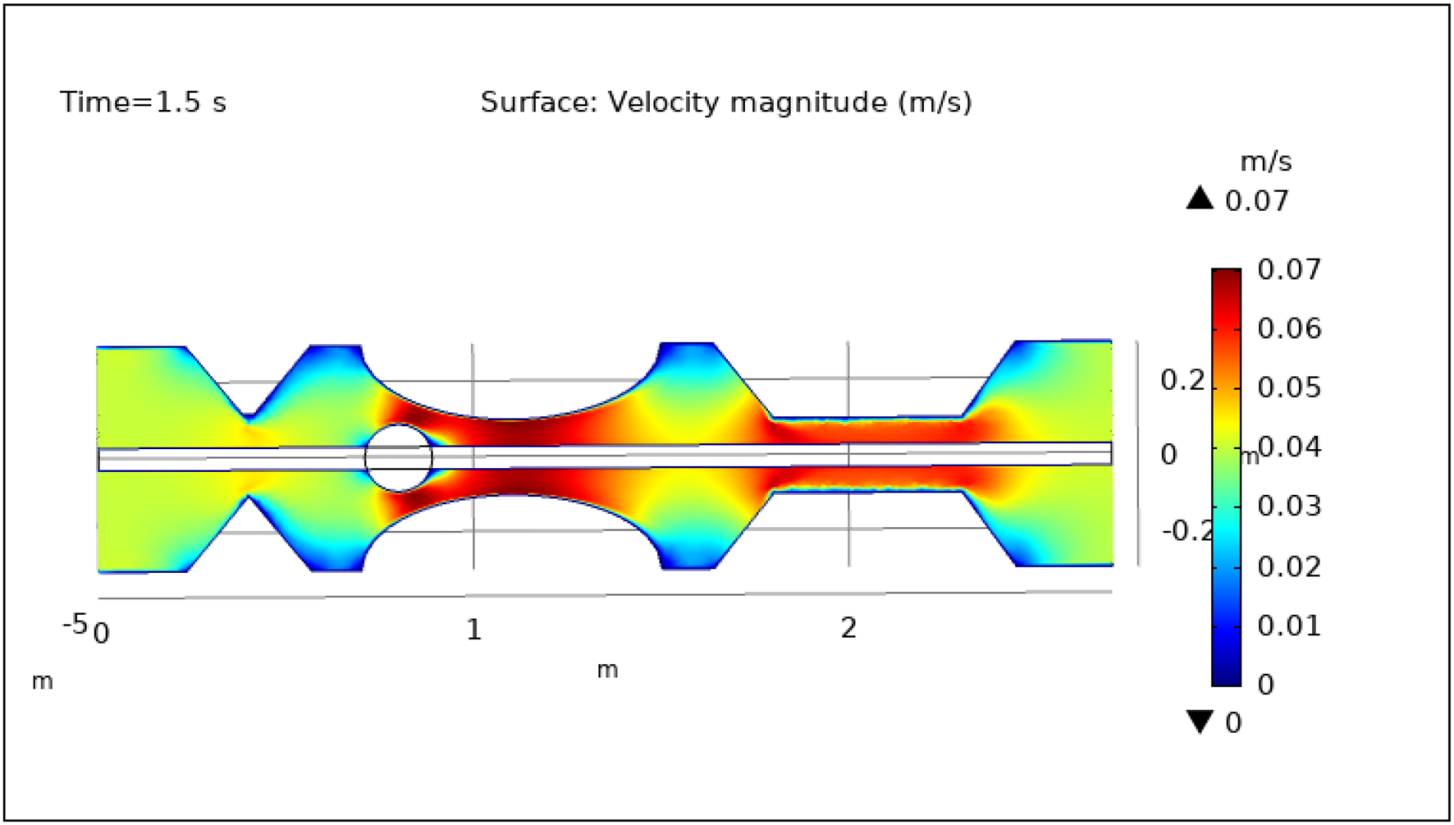
Velocity profile for $t=1.5\text{ s}$.

**Figure 5:**
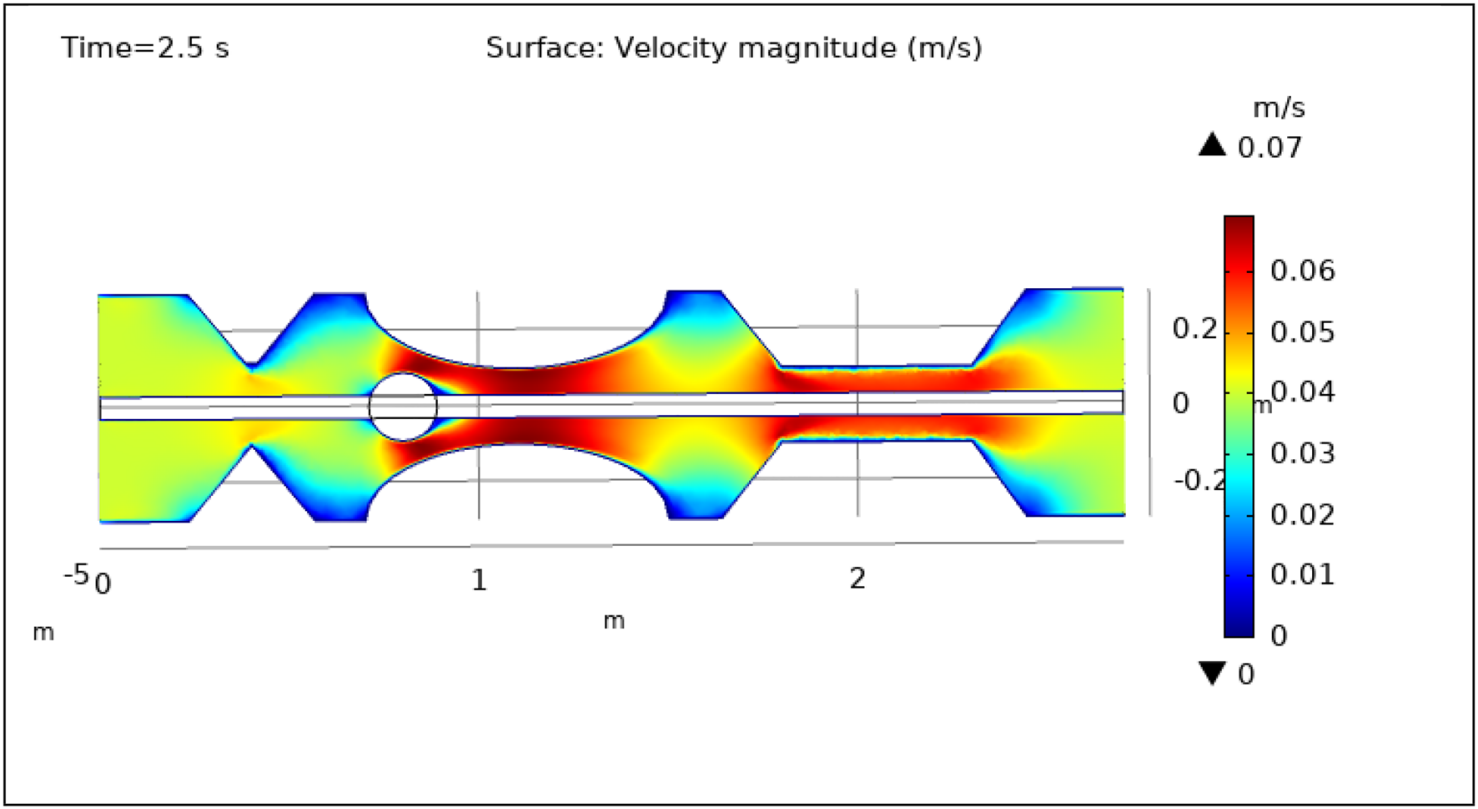
Velocity profile for $t=2.5\text{ s}$.

**Figure 6:**
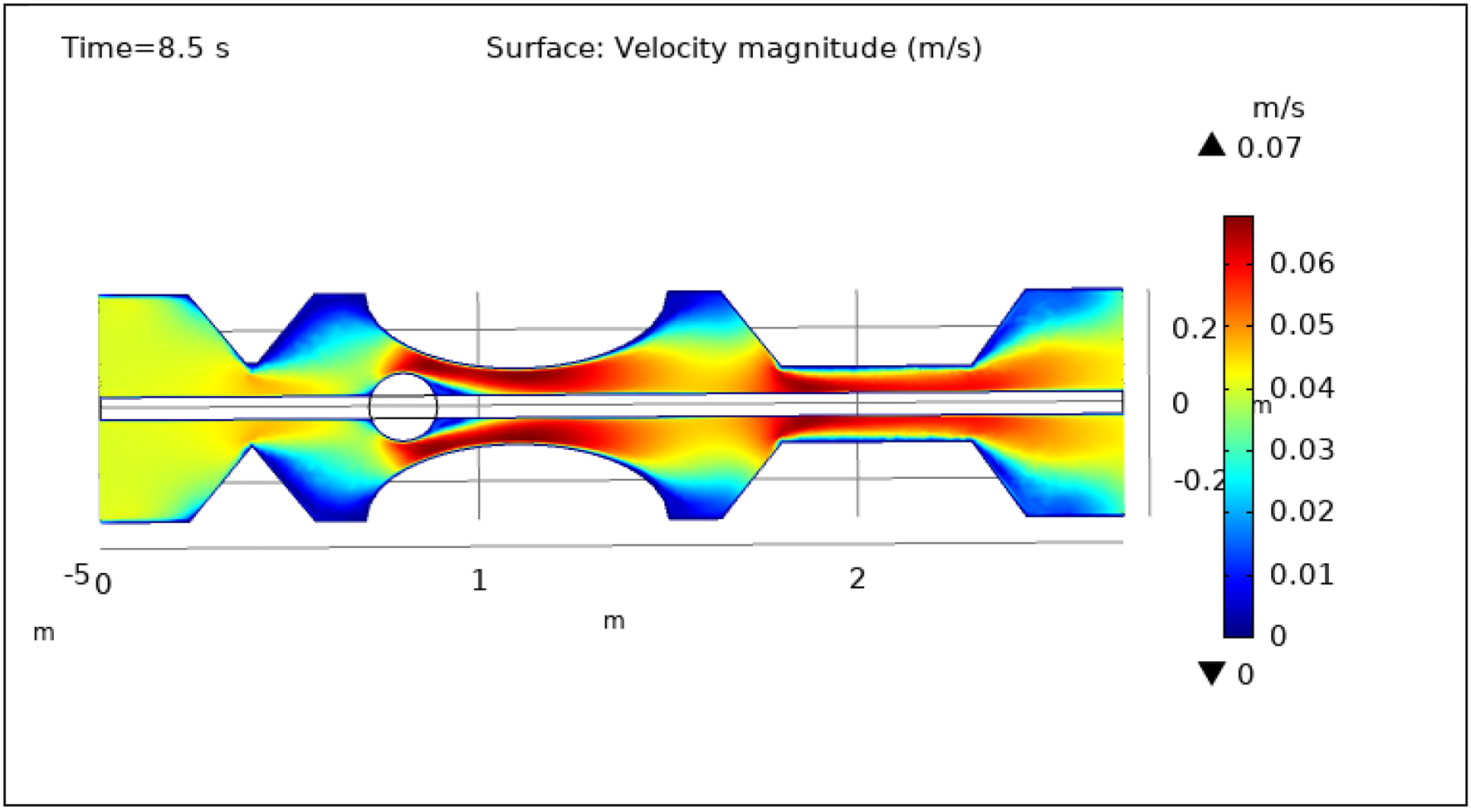
Velocity profile for $t=8.5\text{ s}$.

**Figure 7:**
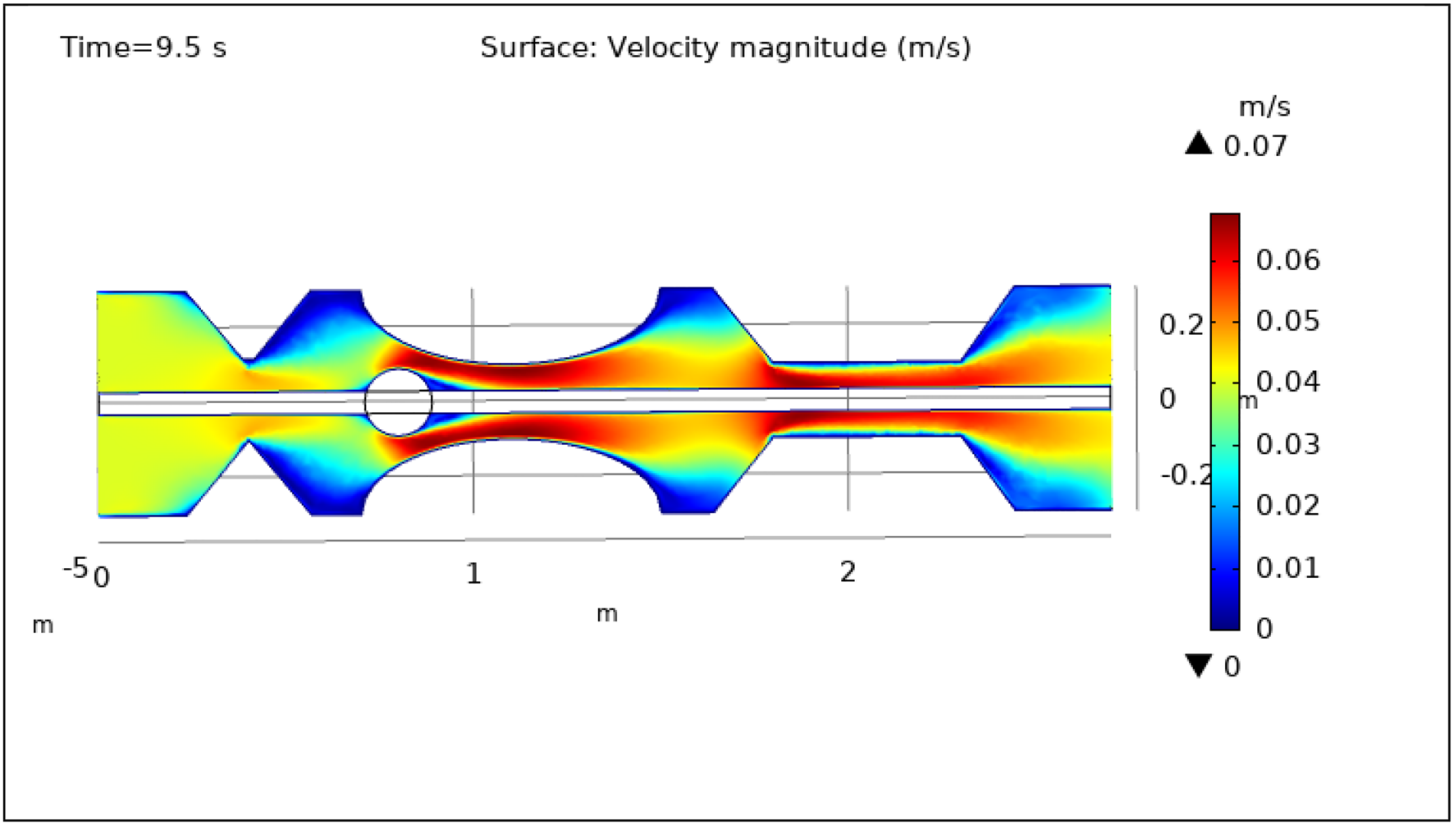
Velocity profile for $t=9.5\text{ s}$.

Figures 3, 4, 5, 6 and 7, delineate the magnitude of the velocity profile at various periods of 0.5 s, 1.5 s, 2.5 s, 8.5 s, and 9.5 s. Figure 3 depicts the surface velocity through three shapes of impedances at the walls, magnetized catheter, and thrombus at the center of the artery for 0.5 s. The maximum velocity at this time is 0.08 ms^−1^ along the artery’s length in the region of elliptical impedance where there is a thrombus and velocity is minimum at the boundary walls as shown by legend. The area where the velocity abruptly upsurges to 0.08 ms^−1^ is due to the blockage at walls and thrombus due to which blood has less space to flow and its speed increases and pressure increases at the boundary which can cause the rupture of the artery. Figure 4 shows the maximum velocity is 0.07 ms^−1^ at 1.5 s. Beyond the stenosis region of the artery, the study identified a transition to a more scattered flow regime. Extensive variations in surface magnitude were observed at both the upper and lower boundaries of the stenosed artery. This observation underscores the significant impact of stenosis on flow patterns and pressures within the artery. The Figure demarcates that velocity is maximum for elliptic shape followed by trapezium and minimum for triangular form. A novel aspect of the study involved the application of an external magnetic field to manipulate the motion of magnetized hybrid nanoparticles, inducing what is commonly referred to as magneto-hydrodynamics (MHD). This process generated micro-scale vortices and secondary flows within the surrounding blood, consequently enhancing blood circulation. These induced flows exhibited the potential to improve blood component mixing, alleviate stagnant zones, and potentially mitigate the adverse effects of stenosis by enhancing blood perfusion. The blood flow improves due to the addition of magnetized hybrid nanoparticles which are injected through a catheter as compared to when we did not deliver nanoparticles in the artery. The magnetic hybrid nanoparticles control the pressure at the boundary walls which reduces the risk of rupture of the artery wall. Figure 5, 6 and 7 delineates the velocity at 2.5 s, 8.5 s, and 9.5 s. The maximum velocity for all these times is 0.07 0.07 ms^−1^ at the center of the artery. An external magnetic field applied to magnetized hybrid nanoparticles can cause fluid movement in the surrounding blood or plasma. This result supports the notion that when blood flows through the tiny segment, blood velocity increases rapidly the pressure imposed on the arterial walls increases. A noteworthy finding was the symmetrical nature of surface velocity and contour velocity patterns observed both above and below the catheter along the entire length of the artery. This symmetry suggests that the presence of the catheter did not disrupt the overall flow symmetry within the artery. In conclusion, these graphs provide valuable insights into the intricate flow dynamics present within arterial segments, particularly in the presence of thrombus and the application of magnetized hybrid nanoparticles. The nanoparticles hold promise as a means to enhance blood flow regulation and reduce the risk of arterial wall rupture. Additionally, the utilization of an external magnetic field to manipulate nanoparticle motion highlights the potential of magneto-hydrodynamics to augment blood circulation and ameliorate complications associated with stenosis. These findings contribute significantly to our understanding of vascular physiology and offer potential avenues for therapeutic interventions in the management of arterial obstructions.

### Surface and contour pressure preeminence

**Figure 8:**
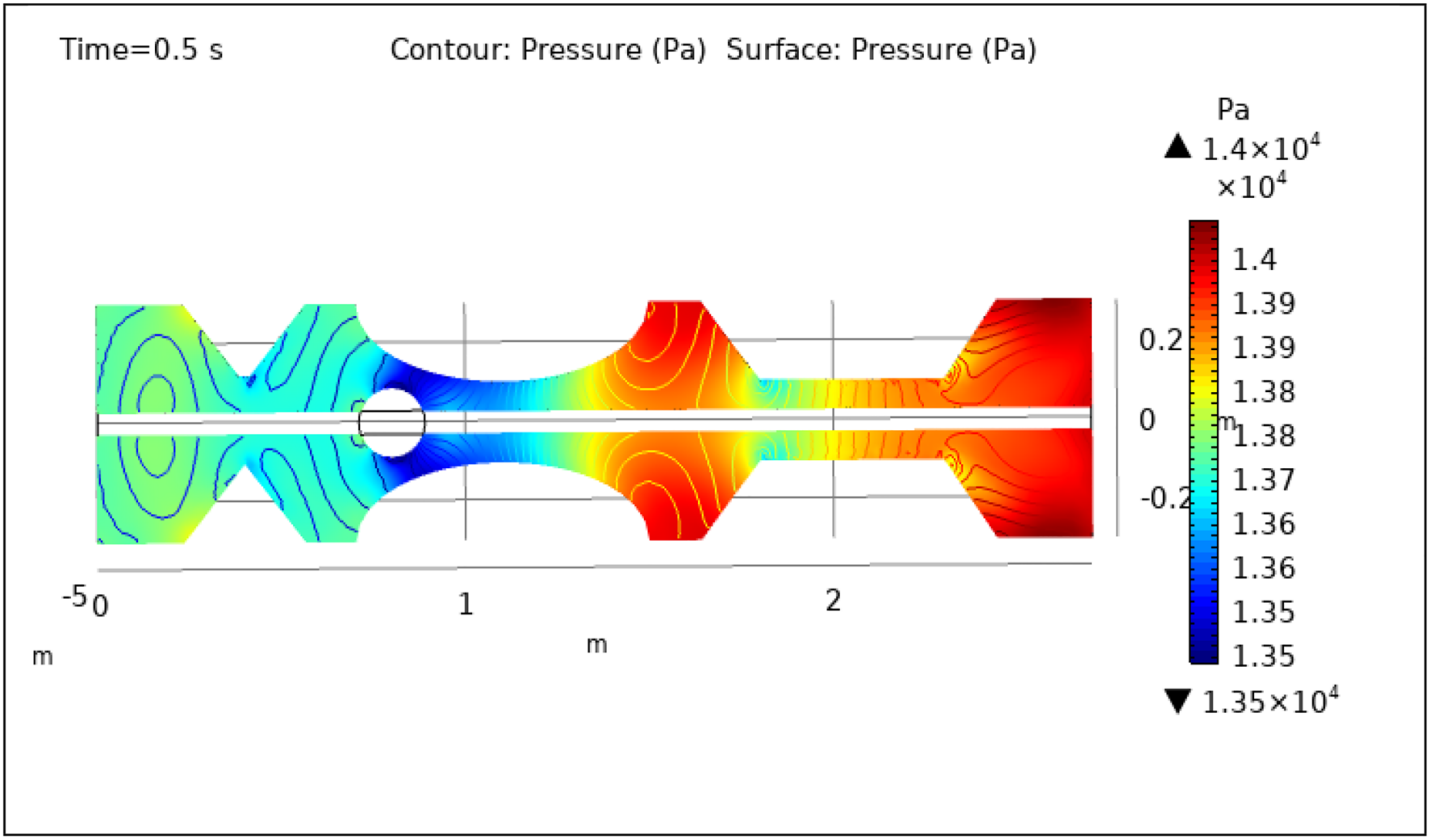
Surface and contour pressure profile for $t=0.5\text{ s}$.

**Figure 9:**
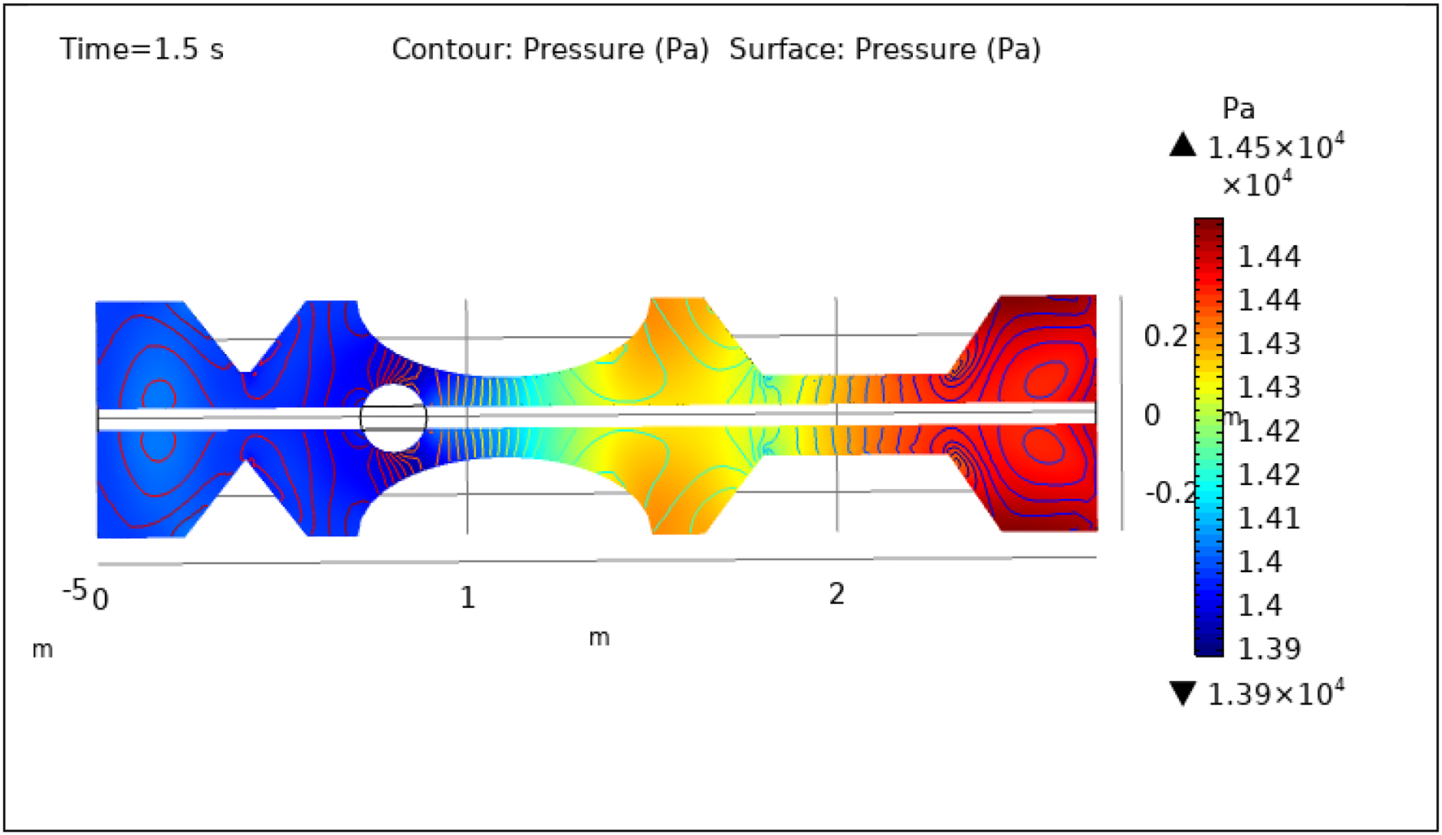
Surface and contour pressure profile for $t=1.5\text{ s}$.

**Figure 10:**
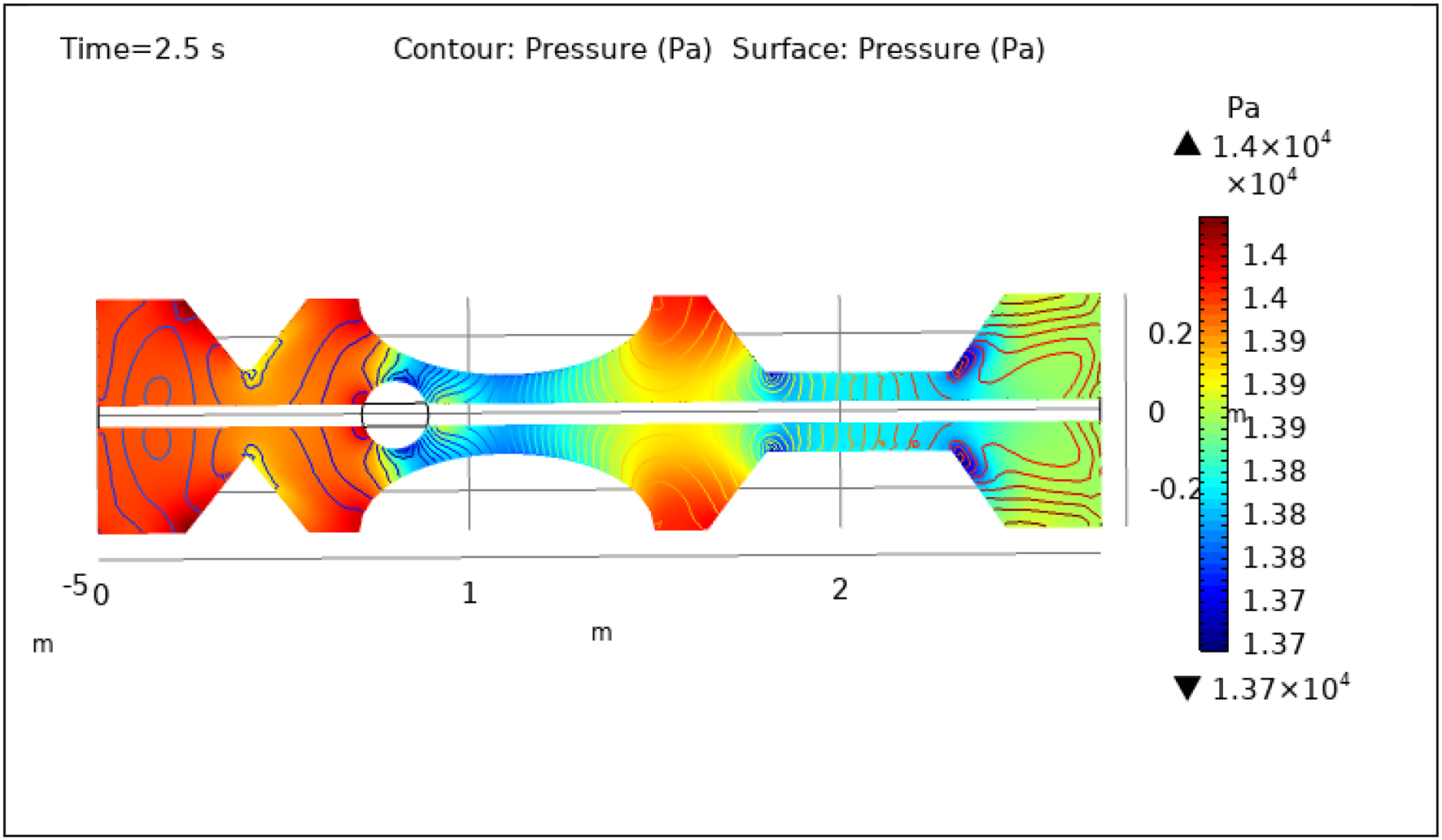
Surface and contour pressure profile for $t=2.5\text{ s}$.

**Figure 11:**
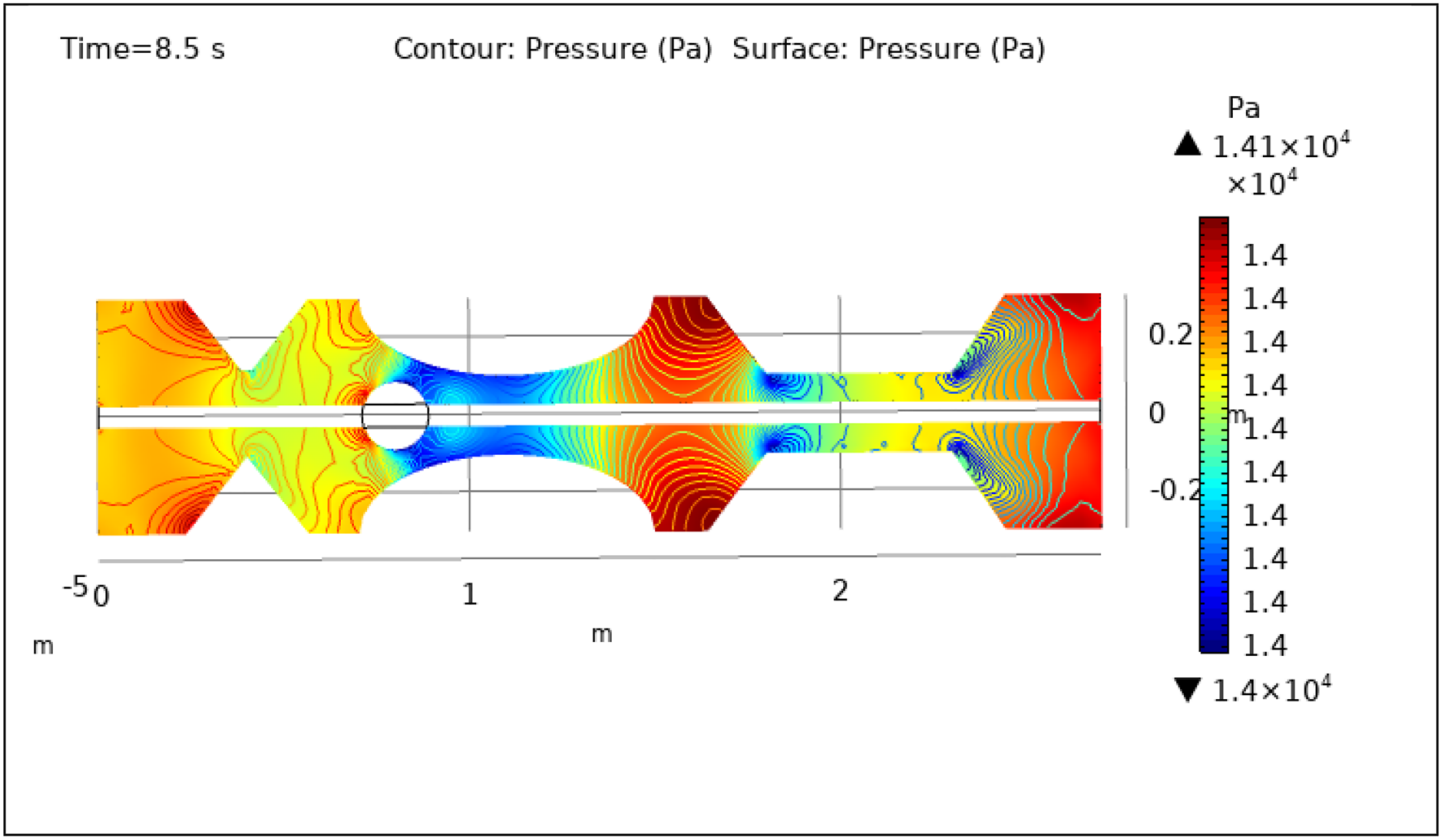
Surface and contour pressure profile for $t=8.5\text{ s}$.

**Figure 12:**
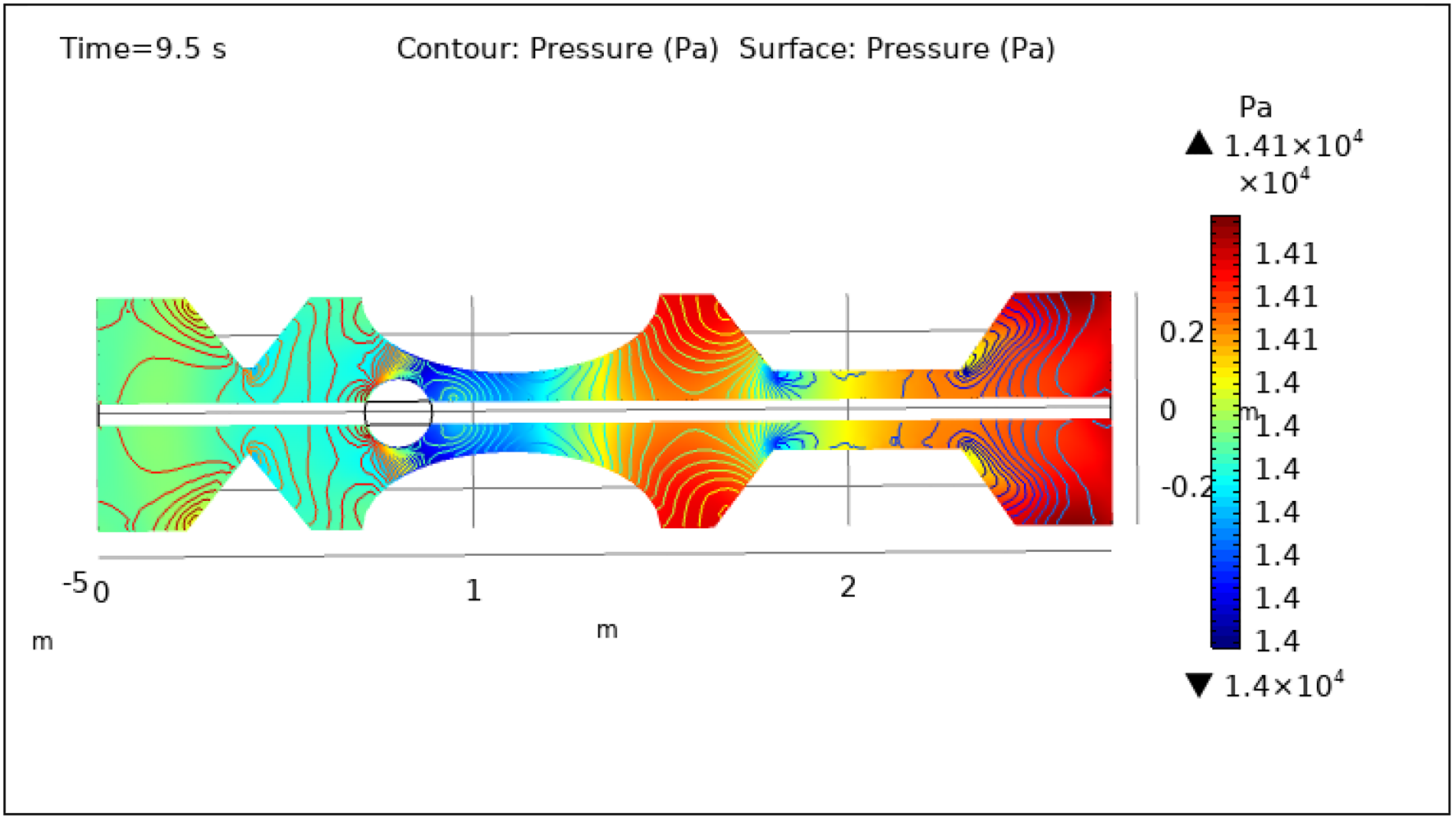
Surface and contour pressure profile for $t=9.5\text{ s}$.

Figures 8, 9, 10, 11 and 12, depict the magnitude of pressure exerted by blood at various periods of 0.5 s, 1.5 s, 2.5 s, 8.5 s, and 9.5 s in 3-D. The pressure endeavor at the walls of a constricted artery owing to fluid activity is depicted for 0.5 s in Fig. 8. It is noted that the pressure gradient is a maximum of $1.4\times {10}^{4}$ pa and a minimum of $1.35\times {10}^{4}$ pa. As can be seen from Fig. 8 the surface pressure and contour pressure are approximately normal all along the artery and it is due to the delivery of magnetized hybrid nanoparticles which is much higher when we did not deliver the nanoparticles. The combination of hybrid nanoparticles, especially those with magnetic properties, along with the application of a magnetic field, shows promise in enhancing the wall shear rate. Fluid flow interactions with the nanoparticles contribute to the rise in wall shear rate. Elevated wall shear rates can lead to improved fluid mixing, increased oxygen and nutrient transfer, and potentially mitigate the formation of blood clots or thrombi. Figure 9 delineates the pressure of hybrid nano-fluid at time 1.5 s. The maximum value of pressure at this period is $1.45\times {10}^{4}$ pa and the minimum is $1.39\times {10}^{4}$ pa throughout the artery which is approximately the normal value of blood pressure in humans. Figures 10, 11 and 12, depict the blood pressure for 2.5, 8.5, and 9.5 s respectively. All these figures suggest that due to the addition of hybrid nanoparticles and magnetic field, pressure decreases at the walls and improves blood flow. The maximum and minimum values for all other times can be seen from legends and also the nature of pressure graphs can be observed in all cases. All the graphs are symmetrical. Pressure values vary concerning position as well as concerning time. The results reveal that this approach has the potential to enhance wall shear rates and promote more uniform pressure distribution along the artery. This could, in turn, facilitate improved fluid mixing, nutrient transport, and potentially mitigate thrombus formation. Importantly, the observed symmetrical pressure profiles and their variation over time highlight the robustness of this approach. These findings hold promise for furthering our understanding of vascular dynamics and the potential development of therapeutic strategies for managing arterial constriction and related complications.

### Surface temperature preeminence

When subjected to an alternating magnetic field, hybrid nanoparticles such as magnetite-gold or magnetite-silver nanoparticles can exhibit outstanding heat-generating capabilities. Magnetic hyperthermia or magnetic nanoparticle hyperthermia is the name given to this phenomenon. When nanoparticles are targeted to stenosed areas and subjected to an external alternating magnetic field, they can create localized heat, perhaps leading to thermal ablation of the stenotic plaque and better blood flow.

**Figure 13:**
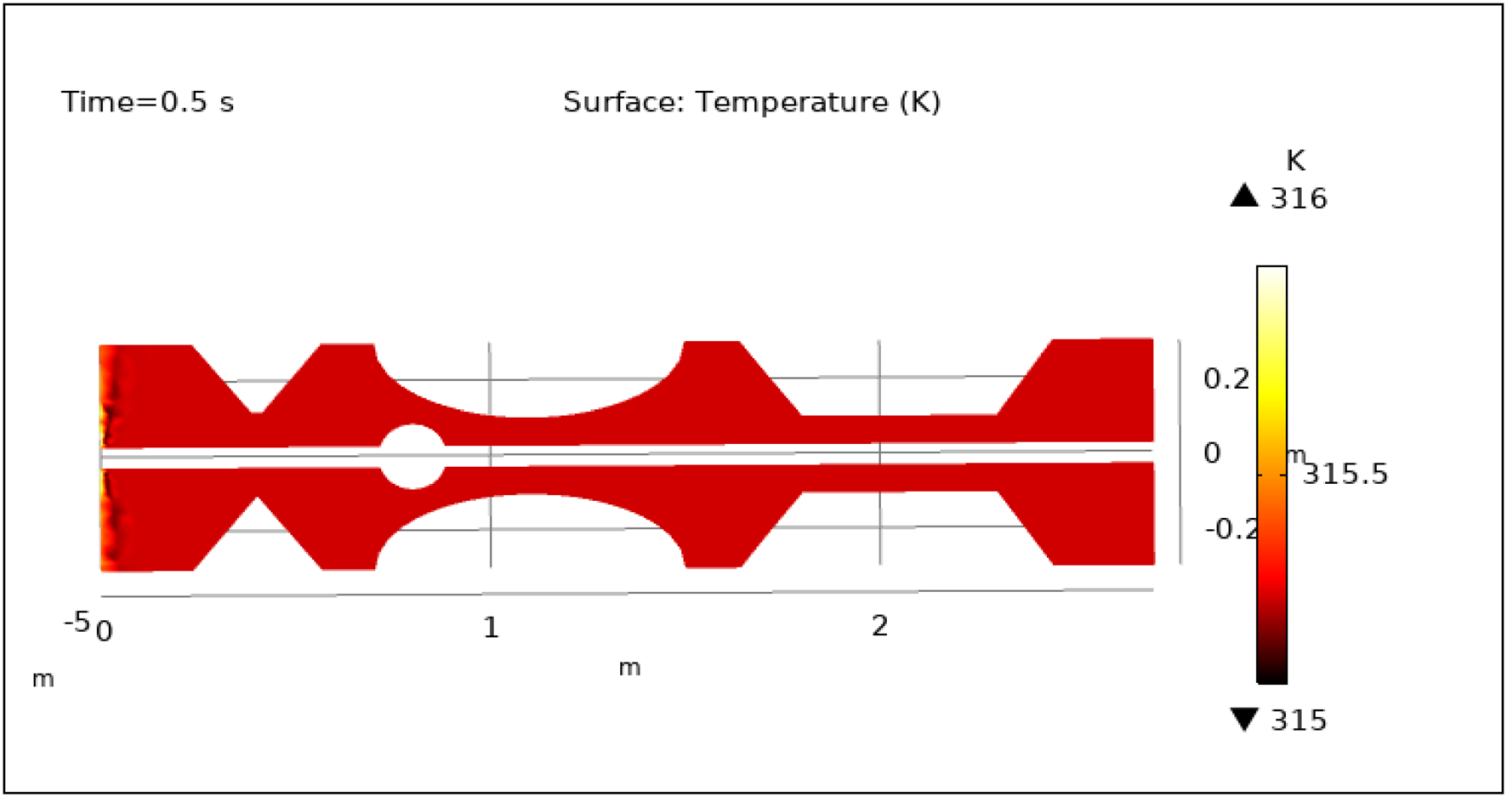
Surface temperature profile for $t=0.5\text{ s}$.

**Figure 14:**
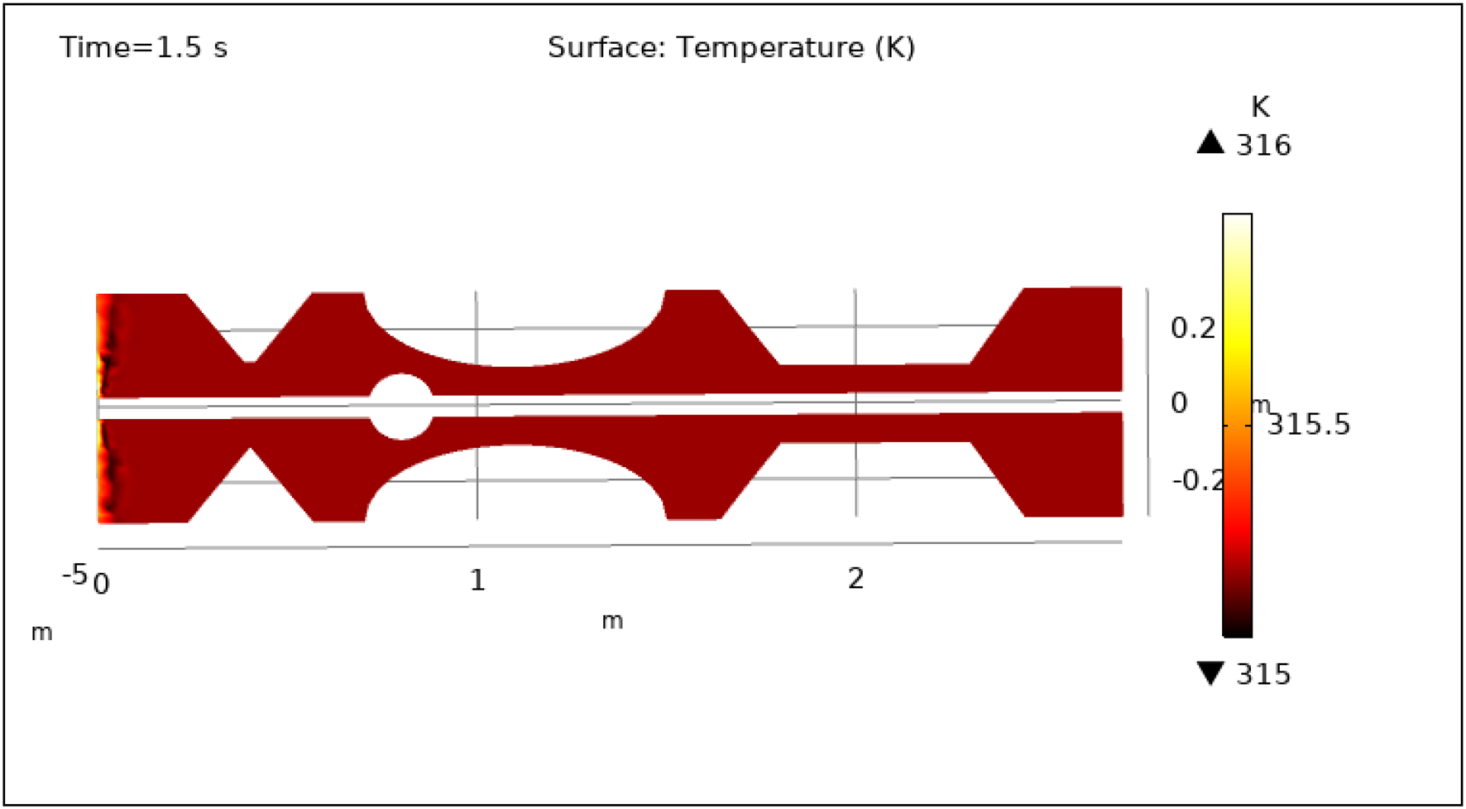
Surface temperature profile for $t=1.5\text{ s}$.

**Figure 15:**
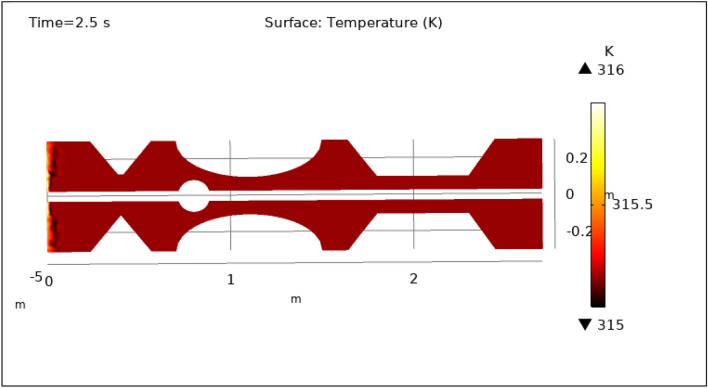
Surface temperature profile for $t=2.5\text{ s}$.

**Figure 16:**
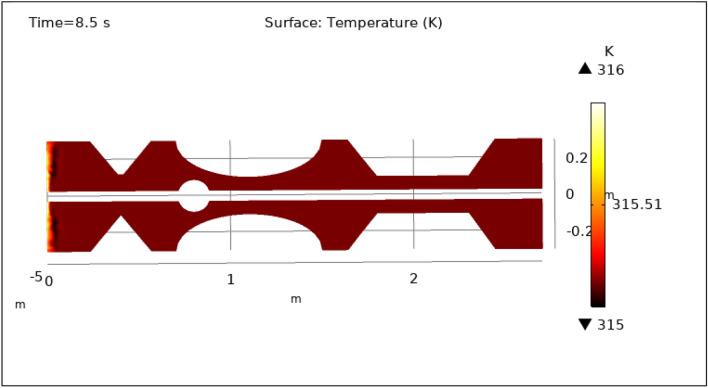
Surface temperature profile for $t=8.5\text{ s}$.

**Figure 17:**
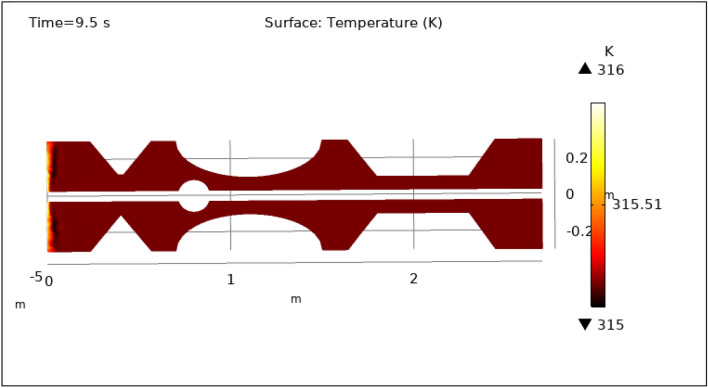
Surface temperature profile for $t=9.5\text{ s}$.

Figures 13, 14, 15, 16 and 17, depict the magnitude of temperature employed by nano-fluid at various periods of 0.5 s, 1.5 s, 2.5 s, 8.5 s, and 9.5 s. Since hybrid nanoparticles are used to control the temperature, we can see in Figs. 13, 14, 15, 16 and 17 that the maximum value of temperature is 316 K and the minimum value is 315 K. There is a very negligible change in the temperature found with respect to time. This is due to the employed magnetic field and delivery of hybrid nanoparticles into the bloodstream. As time increases from 0.5 to 9.5 s the color of the graph gets a bit dark after every step which means temperature is slightly decreasing with time. The maximum temperature is at the inlet otherwise the temperature remains almost constant throughout the stenotic artery. In summary, magnetic hyperthermia utilizing hybrid nanoparticles represents a promising approach to precisely control temperature within stenosed arteries. The study demonstrates stable and controlled temperature profiles, with minimal temporal temperature variation. The application of a magnetic field enhances heat transport and convection, contributing to effective cooling and mitigating the risk of excessive temperature rise within stenotic zones. These findings hold significant potential for the development of therapeutic strategies aimed at improving blood flow and addressing stenotic conditions.

## Description of line graphs

Line graph depicts the performance of blood flow and temperature all along the artery.

**Figure 18:**
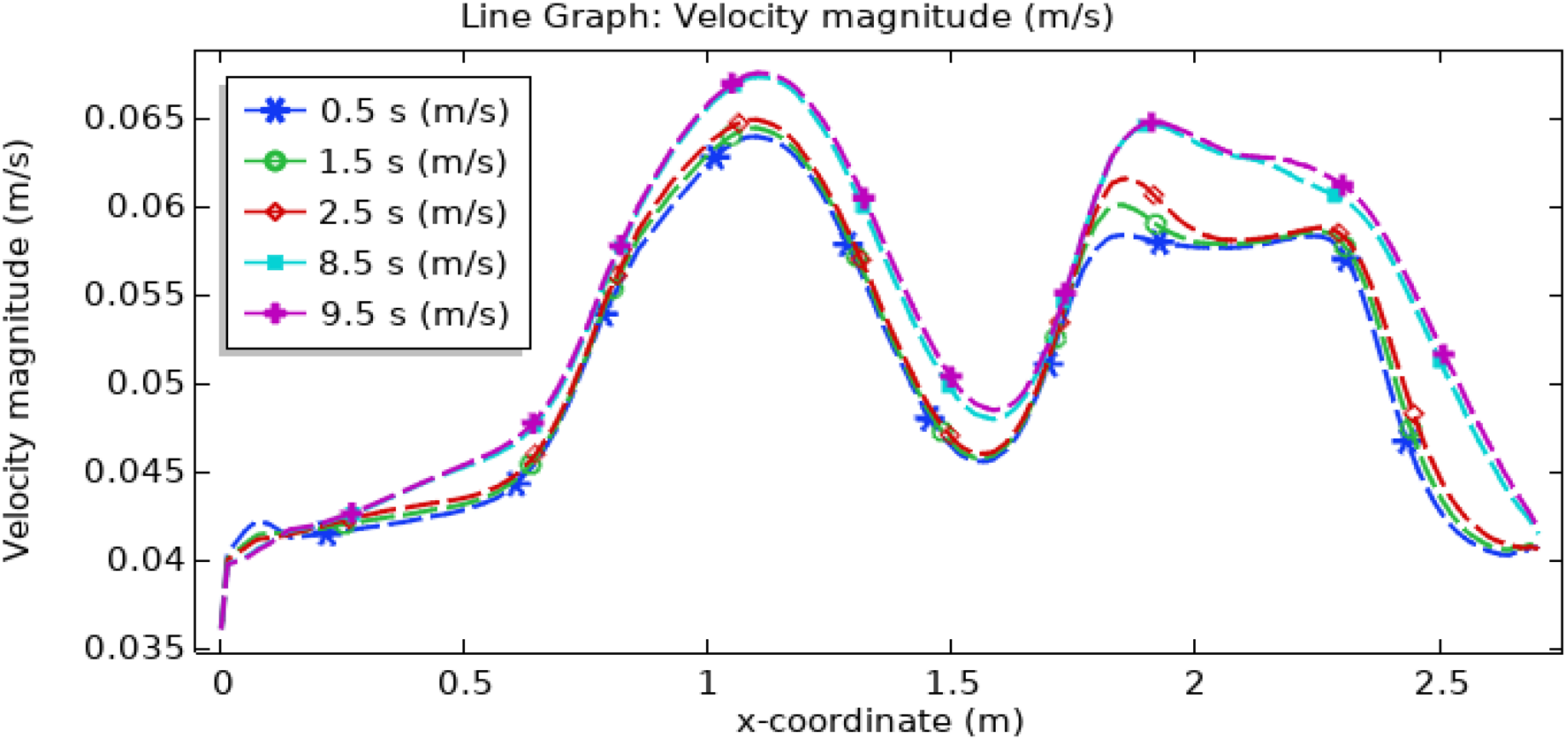
Line graph of blood velocity.

Figure 18 presents a comprehensive visualization of the velocity profile as a function of both position along the x-axis and time within the arterial system. Initially, within the non-stenotic region, blood flow maintains a consistent, physiologically normal velocity. However, as the bloodstream encounters the triangular stenosis, there is a discernible increment in velocity, reaching an approximate value of 0.045 ms^−1^. Upon entering into the domain of the elliptical stenosis and thrombus, the velocity undergoes a significant surge, culminating in its zenith at approximately 0.06 ms^−1^. Within the confines of the trapezoidal stenosis, velocity displays notable variations, ascending to a maximum of 0.06 ms^−1^ during specific time intervals, such as 0.5, 1.5, and 2.5 s. Remarkably, the velocity records slightly elevated levels during time intervals of 8.5 and 9.5 s. In summation, the velocity profile distinctly portrays a direct temporal correlation with velocity along the x-axis. As time unfolds, there is a corresponding augmentation in velocity. The highest velocities manifest within the elliptical stenosis segment, while the lowest velocities are evident in the presence of triangular-shaped stenosis. The legend embedded in the graph impeccably elucidates the temporal evolution of velocity.

**Figure 19:**
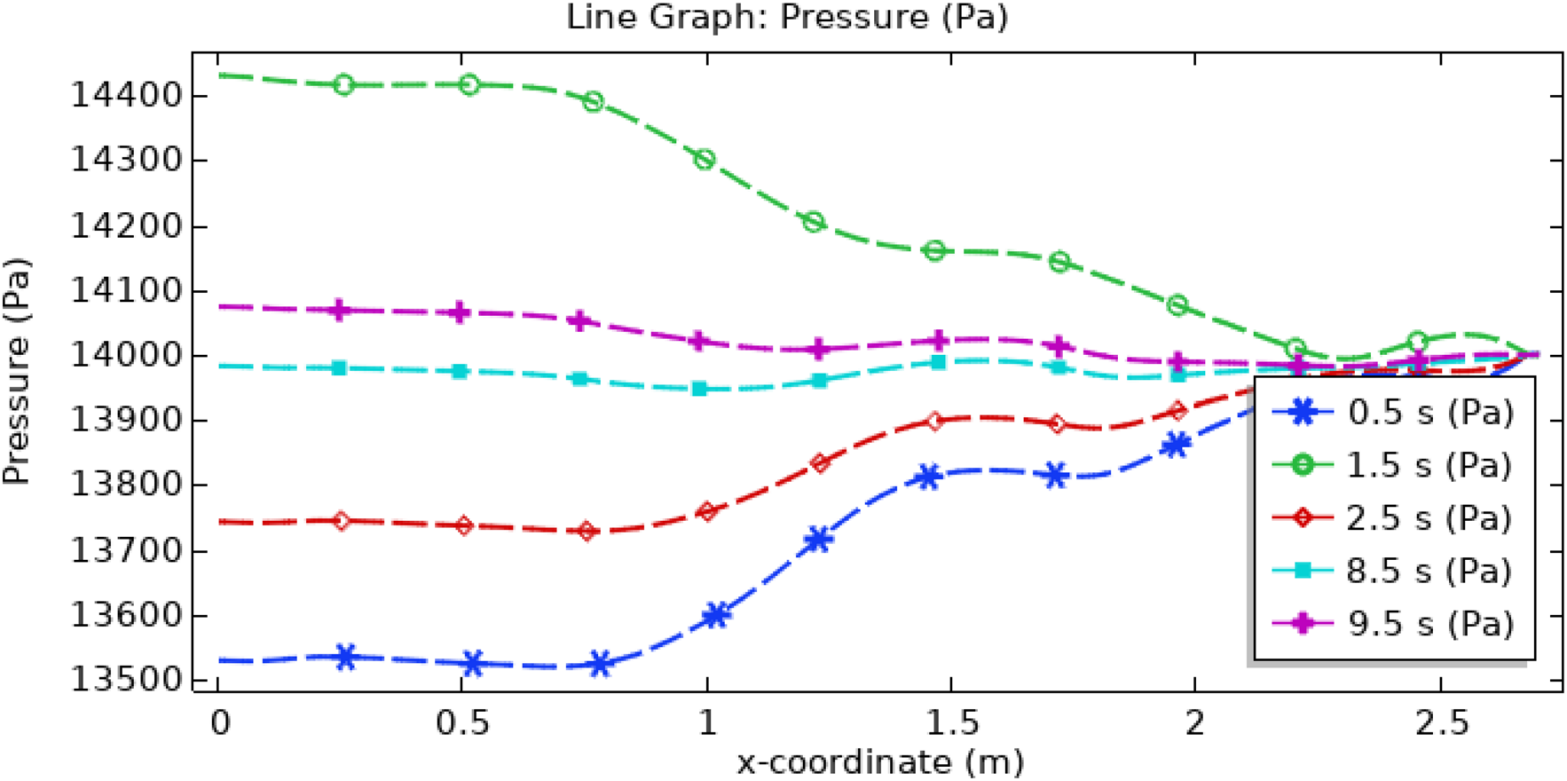
Line graph of blood pressure.

Figure 19 presents the pressure profile within the artery, highlighting the influence of introducing gold and silver hybrid nanoparticles along with a uniform magnetic field. Notably, this combination results in a substantial reduction in pressure, dropping from 14,500 to 13,500 Pa compared to the scenario with the base fluid alone. Throughout the examined time intervals, the inlet of the artery consistently exhibits the highest pressure, with the peak occurring at 1.5 s and the lowest at 0.5 s. As the blood traverses the artery, including regions with thrombus and various stenotic structures, it gradually converges to a nearly uniform pressure level of approximately 14,000 Pa, as depicted in Fig. 19. This graph succinctly illustrates the significant impact of hybrid nanoparticles and magnetic fields on pressure dynamics.

**Figure 20:**
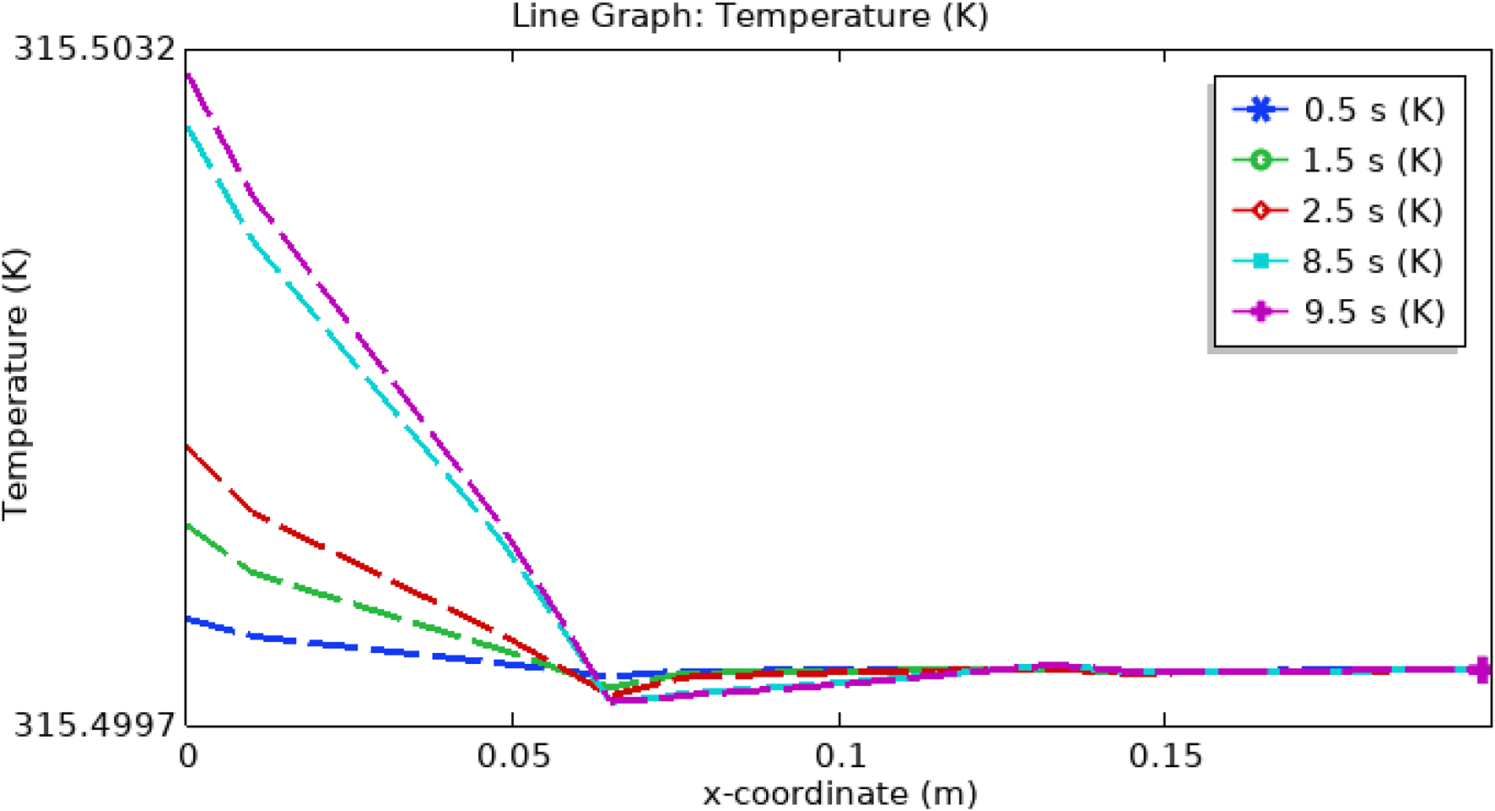
Line graph of blood temperature.

Figure 20 provides a comprehensive depiction of the temperature profile within the artery. Notably, the temperature reaches its maximum value at the inlet for all examined time intervals, as indicated by the color legend. Subsequently, as blood traverses a specific segment of the artery, a noticeable temperature reduction occurs. Beyond this point, the temperature stabilizes and maintains a relatively constant level throughout the entire arterial passage. Furthermore, temporal variations in temperature are evident, as highlighted by the accompanying legend. The most significant temperature fluctuations occur at 9.5 s, followed by 8.5, 2.5, and 1.5 s, with the least variation observed at 0.5 s. In summary, Fig. 20 effectively portrays the temperature dynamics within the artery, with the highest temperatures at the inlet and subsequent stabilization along the arterial length. These temperature changes are also influenced by the passage of time, as indicated by the legend.

**Figure 21:**
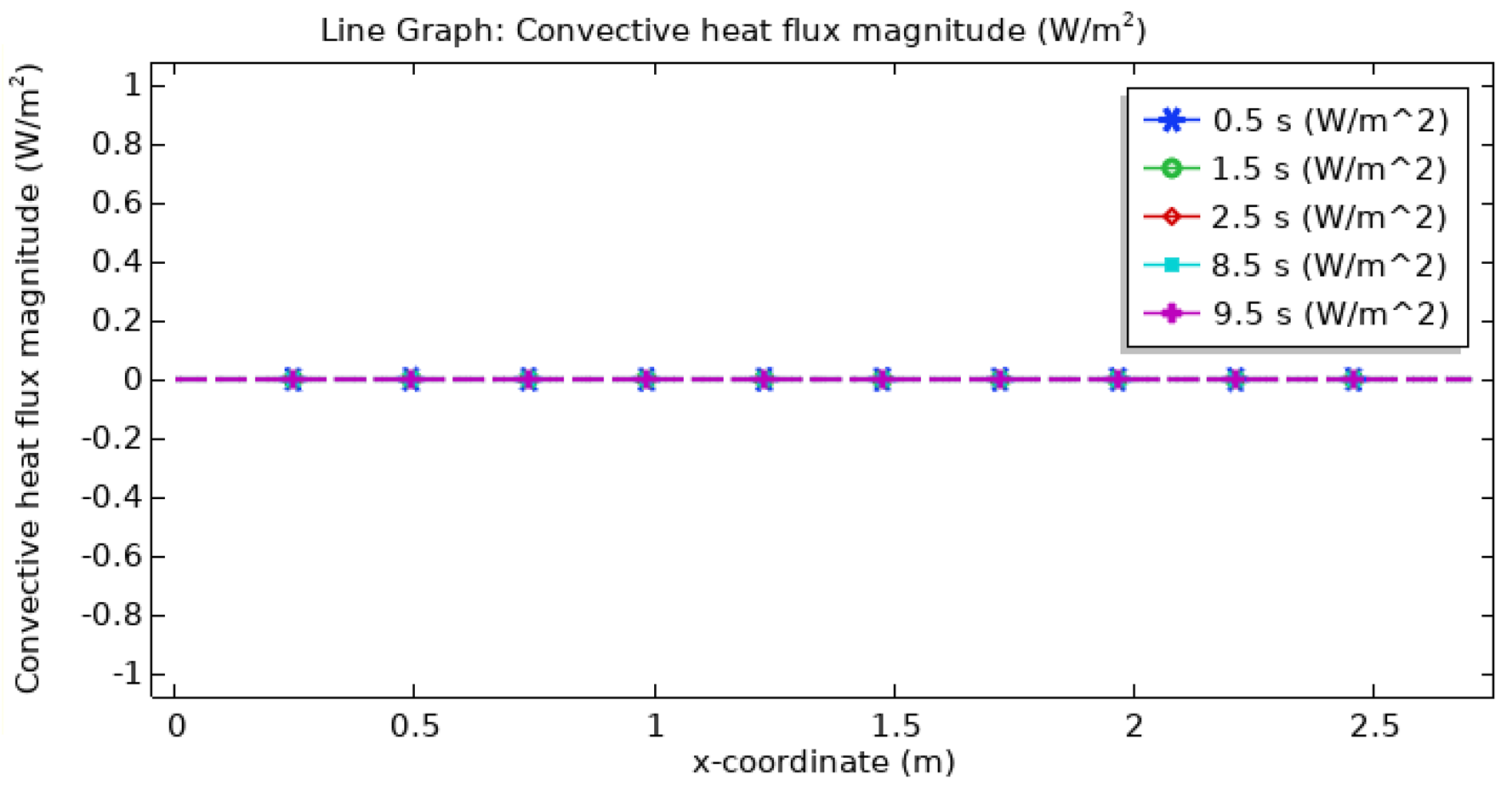
Line graph of convective heat flux.

Figure 21 displays the convective heat flux profile within the artery, offering insights into the heat transfer between a surface and the surrounding fluid or gas at various locations or along a defined pathway. Remarkably, Fig. 21 demonstrates that the convective heat flux remains consistently uniform throughout the entire length of the artery concerning both position and time. This uniformity signifies that there is no significant variation in convective heat flux within the arterial system.

## Concluding remarks

In this study, a mathematical and computational model has been constructed to explore the features of blood flow inoculating silver and gold hybrid nanoparticles and uniform magnetic field in multiple stenosed catheterized arteries with thrombosis. Newtonian properties of blood and the result was determined numerically using the finite element approach (FEM). The catheter is used to regulate the limited flow over the affected artery. It is imperative to remember that in certain situations, magnetic fields can be employed for magnetic medication aiming. The following are some of the key verdicts of the contemporary investigation as revealed by the graphical analysis:

- The introduction of hybrid nanoparticles and the magnetic field had a notable impact on pressure and velocity profiles. A significant reduction in pressure was observed, particularly within stenotic regions. Velocity profiles exhibited complex variations, with the highest velocities occurring in the presence of elliptical stenosis.
- Magnetized hybrid nanoparticles assist in thrombus clearance when blood clots or thrombi are creating blockage or hindering blood flow. These nanoparticles are programmed to adhere to the clot or thrombus, and an external magnetic field is utilized to attract and concentrate them at the location. This localized concentration helps with clot breakdown or dissolution, resulting in increased blood flow.
- The convective heat flux remained remarkably uniform throughout the artery, suggesting consistent heat transfer characteristics along its length.
- Fluid flow and the interaction between the nanoparticles and the blood flow are responsible for the rise in the wall shear rate. Higher wall shear rates can enhance fluid mixing, oxygen, and nutrition transfer, and perhaps reduce the development of blood clots or thrombi.
- Temperature profiles revealed a consistent rise at the artery’s inlet, followed by a gradual decrease along the arterial length. Temperature changes were most pronounced at specific time intervals, indicating transient behavior.
- Magnetic hyperthermia, enabled by hybrid nanoparticles, offers a precise method for temperature control within stenosed regions. This controlled temperature rise can potentially facilitate the thermal ablation of stenotic plaque, ultimately improving blood flow dynamics.
- Exposure to the uniform magnetic field raises arterial pressure drop, which is most noticeable at the optimal flow rate.

It is crucial to emphasize that the efficacy and consequences of utilizing magnetized hybrid nanoparticles to improve blood flow continue to be investigated, and more study is needed to fully understand their potential and limits. The nanoparticles’ exact design, characteristics, and placement, as well as the external magnetic field strength and arrangement, all play key roles in obtaining the necessary blood flow increases.
